# MicroRNAs Regulate the Expression of Genes Related to the Innate Immune and Inflammatory Response in Rabbits Infected with *Lagovirus europaeus* GI.1 and GI.2 Genotypes

**DOI:** 10.3390/ijms25179531

**Published:** 2024-09-02

**Authors:** Ewa Ostrycharz-Jasek, Andrzej Fitzner, Aldona Siennicka, Marta Budkowska, Beata Hukowska-Szematowicz

**Affiliations:** 1 Institute of Biology, University of Szczecin, St. Z. Felczaka 3c, 71-412 Szczecin, Poland; ewa.ostrycharz-jasek@usz.edu.pl; 2 Doctoral School, University of Szczecin, St. A. Mickiewicz 16, 71-412 Szczecin, Poland; 3 Molecular Biology and Biotechnology Center, University of Szczecin, St. Wąska 13, 71-412 Szczecin, Poland; 4 Department of Foot and Mouth Disease, National Veterinary Research Institute-State Research Institute, St. Wodna 7, 98-220 Zduńska Wola, Poland; andrzej.fitzner@piwzp.pl; 5 National Reference Laboratory for Rabbit Hemorrhagic Disease (RHD), St. Wodna 7, 98-220 Zduńska Wola, Poland; 6 Department of Laboratory Diagnostics, Pomeraniam Medical University, St. Powstańców Wielkopolskich 72, 70-111 Szczecin, Poland

**Keywords:** microRNA, *Lagovirus europaeus*/GI.1, GI.2, rabbit hemorrhagic disease (RHD), RHDV, innate immune, inflammation, biomarker, cytokine, rabbits

## Abstract

MicroRNAs (miR) are a group of small, non-coding RNAs of 17–25 nucleotides that regulate gene expression at the post-transcriptional level. Dysregulation of miRNA expression or function may contribute to abnormal gene expression and signaling pathways, leading to disease pathology. *Lagovirus europaeus* (*L. europaeus*) causes severe disease in rabbits called rabbit hemorrhagic disease (RHD). The symptoms of liver, lung, kidney, and spleen degeneration observed during RHD are similar to those of acute liver failure (ALF) and multi-organ failure (MOF) in humans. In this study, we assessed the expression of miRs and their target genes involved in the innate immune and inflammatory response. Also, we assessed their potential impact on pathways in *L. europaeus* infection—two genotypes (GI.1 and GI.2)—in the liver, lungs, kidneys, and spleen. The expression of miRs and target genes was determined using quantitative real-time PCR (qPCR). We assessed the expression of miR-155 (*MyD88*, *TAB2*, *p65*, *NLRP3*), miR-146a (*IRAK1*, *TRAF6*), miR-223 (*TLR4*, *IKKα*, *NLRP3*), and miR-125b (*MyD88*). We also examined biomarkers of inflammation: *IL-1β*, *IL-6*, *TNF-α*, and *IL-18* in four tissues at the mRNA level. Our study shows that the main regulators of the innate immune and inflammatory response in *L. europaeus*/GI.1 and GI.2 infection, as well as RHD, are miR-155, miR-223, and miR-146a. During infection with *L. europaeus*/RHD, miR-155 has both pro- and anti-inflammatory effects in the liver and anti-inflammatory effects in the kidneys and spleen; miR-146a has anti-inflammatory effects in the liver, lungs and kidneys; miR-223 has anti-inflammatory effects in all tissues; however, miR-125b has anti-inflammatory effects only in the liver. In each case, such an effect may be a determinant of the pathogenesis of RHD. Our research shows that miRs may regulate three innate immune and inflammatory response pathways in *L. europaeus* infection. However, the result of this regulation may be influenced by the tissue microenvironment. Our research shows that infection of rabbits with *L. europaeus*/GI.1 and GI.2 genotypes causes an overexpression of two critical acute phase cytokines: *IL-6* in all examined tissues and *TNF-α* (in the liver, lungs, and spleen). *IL-1β* was highly expressed only in the lungs after *L. europaeus* infection. These facts indicate a strong and rapid involvement of the local innate immune and inflammatory response in *L. europaeus* infection—two genotypes (GI.1 and GI.2)—and in the pathogenesis of RHD. Profile of biomarkers of inflammation in rabbits infected with *L. europaeus*/GI.1 and GI.2 genotypes are similar regarding the nature of changes but are different for individual tissues. Therefore, we propose three inflammation profiles for *L. europaeus* infection for both GI.1 and GI.2 genotypes (pulmonary, renal, liver, and spleen).

## 1. Introduction

Rabbit hemorrhagic disease (RHD) is caused by *L. europaeus* [1,2]. There are four genotypes within the *Lagovirus* genus (GI.1, GI.2, GI.3 and GI.4) [1]. The *L. europaeus*/GI.1 genotype includes four variants (GI.1a, GI.1b, GI.1c, and GI.1d), with the GI.1a variant causing an acute, inflammatory, highly contagious, and fatal disease with a mortality rate of 90–100% [3]. However, the *L. europaeus*/GI.2 genotype in infected rabbits causes a disease with variable mortality (from 50% to 80%, depending on the strain) and clinical presentation from sudden death in the hyperacute form to subacute or chronic disease [4,5]. The main target tissues of *L. europaeus* are the liver, lungs, spleen, and kidneys. However, where the most significant pathological changes are observed, the liver replicates the virus [3]. During infection, the liver becomes enlarged with a distinct lobular pattern. Moreover, increased virus-induced hepatocyte loss is observed in the liver [3,4]. The main changes in RHD are acute liver, spleen, lung, and kidney inflammation. Other pathological changes in RHD include spleen and kidney enlargement and pulmonary edema [3]. Inflammatory foci rich in neutrophils, T and B lymphocytes are found in the liver, lungs, spleen, and kidneys, and the activation of Kupffer cells occurs in the liver and alveolar macrophages in the lungs [3,4]. However, coagulation disorders, microthrombi formation, and massive disseminated intravascular coagulation (DIC) influence the occurrence of hemorrhages [3,5,6]. Studies have shown that necrosis is another critical factor in the pathogenesis of RHD [7]. As well as increased apoptosis, not only in the liver but in all infected tissues [8,9,10,11,12,13,14,15,16,17,18] and apoptosis of T and B lymphocytes in the liver and spleen and granulocytes and lymphocytes in peripheral blood [13,19,20,21], there is also increased oxidative stress [10,16,22].

In our recent studies [16,23,24,25], we indicated that another element in the pathogenesis of RHD are microRNAs (miRs), representing a subclass of small non-coding, single-stranded RNAs that, by binding to mRNA, post-transcriptionally regulate gene expression [26]. Our reports [16,24] are the first to present the regulatory effects of miRs on apoptosis, oxidative stress, and inflammation genes in *L. europaeus* infection—two genotypes (GI.1 and GI.2)—in four rabbit tissues (liver, lung, kidneys, and spleen). Our studies provide new data that are critical for understanding the pathogenesis of RHD caused by *L. europaeus*—two genotypes (GI.1 and GI.2)—concerning the molecular regulation of apoptosis and oxidative stress by miRs (as two essential biological processes in infectious viruses). Increased expression of miR-132 and miR-122 through the regulation of *Nrf-2*, *Bach1*, and *HO-1* genes regulates oxidative stress in the pathogenesis of RHD and affects tissue damage in *L. europaeus*-infected animals. Additionally, changes in the expression levels of proapoptotic (miR-16b and miR-34a) and antiapoptotic (miR-21) miRs and their impact on apoptosis-related target genes (*Bcl-2*, *PTEN*, *SIRT1/p53*) may intensify apoptosis, contributing to more severe disease and the death of animals [16]. Our findings show that miR-21, miR-16b, and miR-34a regulate three apoptosis pathways. Meanwhile, miR-122 and miR-132 are involved in two oxidative stress pathways [16].

The innate and adaptive immune response, including peripheral blood leukocytes and systemic inflammation, also plays an essential role in the pathogenesis of RHD. 

The innate immune response after infection of rabbits with over thirty different strains of *L. europaeus* was mainly manifested by variable phagocytosis activity by neutrophils and the killing capacity of leukocytes through the activity of enzymes with killing/antiviral properties—myeloperoxidase (MPO) and lysozyme (LZM) [20,27,28,29,30,31,32,33]. The participation and intensity of these processes depended on the *L. europaeus* variant/strain. Moreover, in the infected organs (mainly the liver, lungs, kidneys, and spleen), infiltrates rich in leukocytes (neutrophils, T and B cells) and an increase in the inflammatory biomarker miR-155 are observed [23,34,35]. It has also been shown that during *L. europaeus* infection, the levels of both pro- and anti-inflammatory cytokines increase in tissues (liver and spleen) and peripheral blood leukocytes. These cytokines include IL-1, IL-6, IL-8, IL-10, TNF-α, TNF-β, IFN-γ, and granulocyte-macrophage colony-stimulating factor (GM-CSF) [36,37,38,39,40,41]. Additionally, O’Toole et al. [42] indicate that *L. europaues* infection/GI.2 genotype drives the pathogenesis of RHD through a cytokine storm. Hepatocytes excessively produce TNF-α, IL-1β and IL-6, leading to hypercoagulability. In 2024, Yu et al. [43] showed that pro-inflammatory cytokines (IL-1α, IL-6, IL-8, IL-22) and chemokines (CCL2, CXCL9), involved in inflammation, are significantly increased in the spleen in the late stages of *L. europaeus* infection/GI.2 genotype. These data suggests that *L. europaeus*/GI.2 genotype (RHDV2) infection may induce dysregulation of the cytokine network and weaken the spleen’s resistance to viral infection, leading to inflammatory disorders. 

However, the adaptive immune response was characterized by variable activity of lymphocytes with different phenotypes. These phenotypes included T (CD5+), Th (CD4+), Tc (as CTLs) (CD8+),Tregs (CD25+), and B lymphocytes (CD19+). This variability indicates an impaired immune response, sometimes resulting in the complete loss of effector cells in a short time and changes in the total amount of immunoglobulins [20,28,29,44,45].

MiRs are also involved in the host’s immune and inflammatory response to an inflammatory stimulus [46]. The role of miRs in the innate immune and inflammatory response context may be twofold [46,47,48]. On the one hand, the expression of miRs may be directly regulated by innate immune responses (since intracellular levels of different miRs are initially regulated at the transcriptional level by transcription factors dependent on the cell, tissue type, and environmental stimuli) [46]. On the other hand, miRs can regulate critical genes of innate immunity, among others: miR-155 regulated *MyD88* (Myeloid differentiation primary response protein MyD88), *TAB2* (GF-beta-activated kinase 1 and MAP3K7-binding protein 2), the *p65* subunit of NF-ĸβ (Transcription factor p65), and the *NLRP3* inflammasome (NACHT, LRR and PYD domains-containing protein 3); miR-146a regulated *IRAK1* (Interleukin-1 receptor-associated kinase 1), and *TRAF6* (TNF receptor-associated factor 6); miR-223 regulated *TLR4* (Toll-like receptor 4), *IKKα* (Inhibitor of nuclear factor kappa-B kinase subunit alpha), and the *NLRP3* inflammasome (NACHT, LRR and PYD domains-containing protein 3); and miR-125b regulated *MyD88*, thereby regulating innate immune and inflammatory responses (becoming pro-inflammatory or anti-inflammatory/or even both) [46,47,48,49]. Researchers have identified several dozen miRs involved in regulating the innate immune and inflammatory response, with the well-described being miR-155, miR-146a, miR-223, and miR-125b [46,47,48,49,50,51,52].

So far, apart from our studies [16,23,24,25,53], there is a lack of information on the molecular signatures of regulatory interactions between miRs and biological processes occurring in *L. europaeus* infection/RHD pathogenesis. There is a lack of studies on the molecular regulatory interactions between miRs and the innate immune and inflammatory response in *L. europaeus* infection. Therefore, this issue is the aim of this study. The study assessed the expression of miRs and their target genes involved in the regulation of the innate immune and inflammatory response, as well as presenting their potential impact on the pathways in *Lagovirus europaeus* infection—two genotypes (GI.1 and GI.2)—of different virulence in four tissues (liver, lungs, kidneys, and spleen). Based on in-silico analysis and previous literature data [24,25,46], we selected known miRs and target genes involved in the regulation of the innate immune and inflammatory response (essential in the TLR4-MyD88, NF-κB, NLRP3 inflammasome signaling pathway): miR-155 (*MyD88*, *TAB2*, *p65*, *NLRP3*), miR-146a (*IRAK1*, *TRAF6*), miR-223 (*TLR4*, *IKKα*, *NLRP3*), and miR-125b (*MyD88*). Also, we examined biomarkers of inflammation at the mRNA level: *IL-1β*, *IL-6*, *TNF-α*, and *IL-18* in the examined tissue.

Understanding the above molecular interactions has diagnostic potential (search for potential molecular biomarkers of inflammation/disease) and therapeutic potential (modulation of miR-dependent pathways, e.g., NF-ĸB and NLRP3 inflammasome) in the course of acute liver failure (ALF) and organ dysfunction in multi-organ failure (MOF) of a viral etiology that we encounter during *Lagovirus europaeus* infection.

## 2. Results

### 2.1. MiRs Expression Levels and Its Downstream Targets Involved in Innate Immune and Inflammatory Responses in Four Tissues in Rabbits during Lagovirus europaeus/GI.1 and GI.2 Genotype Infection 

We analyzed the expression of miRs (miR-155, miR-146a, miR-223, and miR-125b) and its downstream targets involved in the innate immune and inflammatory response in four tissues (liver, lung, spleen, and kidney) in rabbits infected with *L. europaeus*/GI.1 and GI.2 genotypes.

#### 2.1.1. Liver

**Figure 1:**
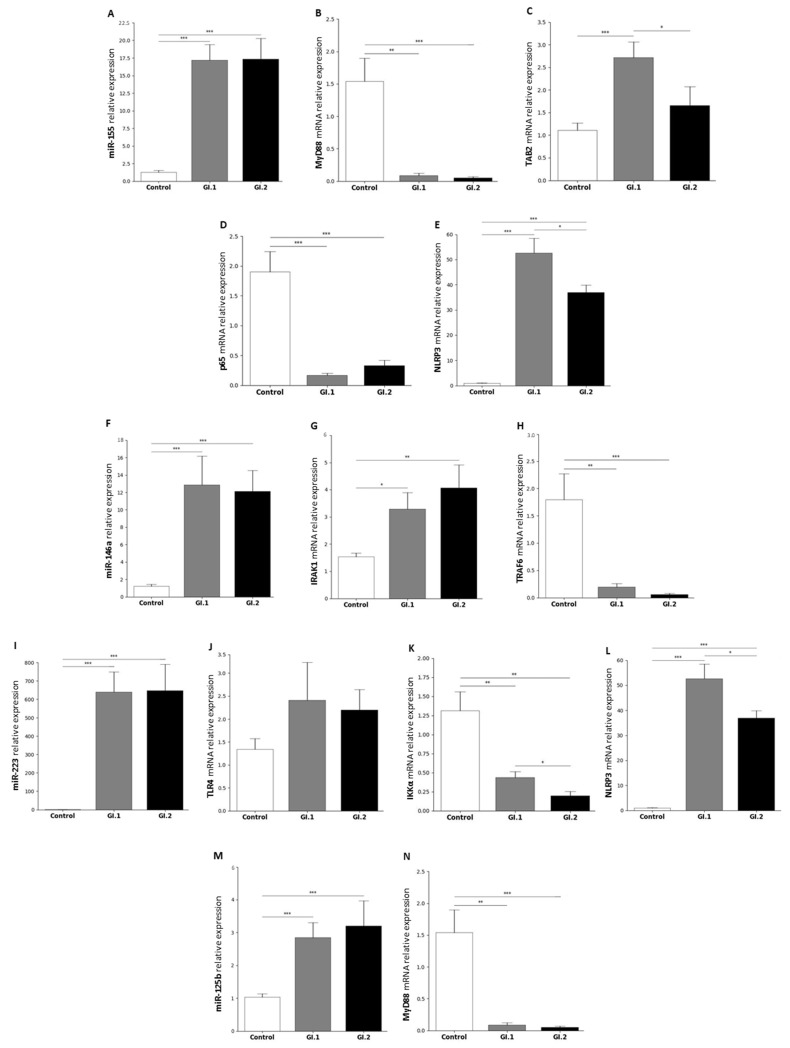
Relative expression of miRs and downstream targets: miR-155 (**A**) (*MyD88* (**B**), *TAB2* (**C**), *p65* (**D**), *NLRP3* (**E**)), miR-146a (**F**) (*IRAK1* (**G**), *TRAF6* (**H**)), miR-223 (**I**) (*TLR4* (**J**), *IKKα* (**K**), *NLRP3* (**L**)), miR-125b (**M**) (*MyD88* (**N**)) in the liver during rabbits infection with *Lagovirus europaeus*—two genotypes (GI.1 (*n* = 10) and GI.2 (*n* = 10)) and controls (*n* = 10). The expression of all genes is normalized to an endogenous reference (miR-103a for all tested miRs and 18S rRNA for other genes) and presented as a relative fold change to controls according to the comparative Ct method (2^−ΔΔCt^). The miR and target gene levels were evaluated using real-time PCR. Data were compared with the one-way ANOVA or the ANOVA Kruskal–Wallis test. The *t*-test, or Mann–Whitney U test, assessed the differences in parameter concentrations. *p*-values below 0.05 were considered statistically significant. Bars indicate the mean ± standard error of the mean (SEM), * *p* < 0.5, ** *p* < 0.01, and *** *p* < 0.001.

In the liver after infection with *L. europaeus*, a very similar increased miR-155 expression was observed (13.6-fold change vs. control, *p* < 0.001 and 13.7-fold change vs. control, *p* < 0.001 for GI.1 and GI.2, respectively; Figure 1A). In the case of infection with *L. europaeus*/GI.1 and GI.2 genotypes, upregulation of miR-155 was accompanied by decreased levels of *MyD88* (17.5-fold reduction vs. control, *p* = 0.01 and 32-fold reduction vs. control, *p* < 0.001, respectively; Figure 1B) and *p65* (11.5-fold reduction vs. control, *p* < 0.001 and 5.7-fold reduction vs. control, *p* < 0.001, respectively; Figure 1D). However, in the case of *TAB2*, the change in expression was noted only during infection with the GI.1 genotype and was upregulated by a 2.4-fold change (*p* < 0.001) compared to the control (Figure 1C). However, compared to the GI.2 group, in the GI.1 group, the expression was a higher 1.5-fold change (*p* = 0.03; Figure 1C). Our research demonstrated that in the liver, an increase in miR-155 was accompanied by overexpression of the NLRP3 inflammasome by a 53.6-fold change for the GI.1 group compared to the control (*p* < 0.001) and a 37.7-fold change for the GI.2 group compared to the control (Figure 1E). Additionally, the expression of *NLRP3* showed a 1.4-fold change higher in the case of GI.1 infection than in the GI.2 infected group (*p* = 0.03; Figure 1E).

The expression of miR-146a, like miR-155, was enhanced in both infected groups. Compared to the control group during *L. europaeus*/GI.1 infection, the increase in expression of miR-146a was a 10.5-fold change (*p* < 0.001); however, during infection with genotype GI.2, the expression increase was a 9.9-fold change (*p* < 0.001) (Figure 1F). Upregulation of miR-146a was associated with a significant downregulation of *TRAF6* (9-fold change, *p* = 0.003 for GI.1 vs. control and 30.6-fold change, *p* < 0.001 for GI.2 vs. control; Figure 1H). *IRAK1* overexpression was noted during infection of GI.1 and GI.2 (2.1-fold change, *p* = 0.01 vs. control and 2.6-fold change, *p* = 0.001 vs. control; Figure 1G). 

The highest increase in expression among miRs in the liver was observed in the case of miR-223. Compared to the control upregulation of miR-223, it was a 816-fold change in the GI.1 group (*p* < 0.001) and 826-fold change in the GI.2 group (*p* < 0.001) (Figure 1I). No statistically significant change was observed in the expression level of *TLR4*, a target gene of miR-223, in the GI.1 and GI.2 groups (Figure 1J). Upregulation of miR-223 was associated with decreased *IKKα* levels in both infected groups compared to the control (3-fold change, *p* = 0.003 for GI.1 and 6.7-fold change, *p* = 0.002 for GI.2; Figure 1K). However, during infection with the *L. europaeus*/GI.1 genotype, the *IKKα* expression level was a 2.3-fold change higher (*p* = 0.02) than the GI.2 genotype (Figure 1K). In the case of another miR-223 target, the *NLRP3* inflammasome, an increase was observed (53.6-fold change for GI.1 vs. control, *p* < 0.001 and 37.7-fold change for GI.2 vs. control, *p* < 0.001; Figure 1L). Additionally, the expression of *NLRP3* was a 1.4-fold change higher in GI.1 compared to the GI.2 genotype (*p* = 0.03) (Figure 1L). 

In the liver also, miR-125b was significantly higher during infection of both L. europaeus genotypes compared to the control (2.7-fold change, *p* < 0.001 for GI.1 and 3-fold change, *p* < 0.001 for GI.2; Figure 1M). This fact was accompanied by decreased *MyD88* expression levels (17.5-fold reduction vs. control, *p* = 0.01 and 32-fold reduction vs. control, *p* < 0.001; Figure 1N).

#### 2.1.2. Lung

**Figure 2:**
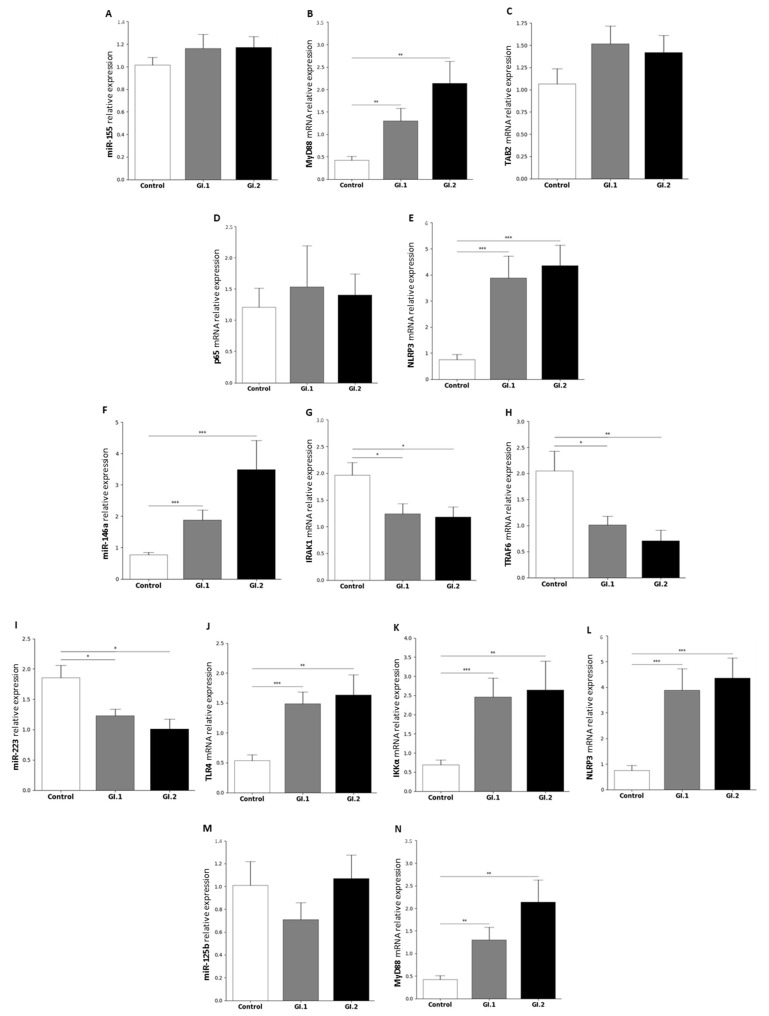
Relative expression of miRs and downstream targets: miR-155 (**A**) (*MyD88* (**B**), *TAB2* (**C**), *p65* (**D**), *NLRP3* (**E**)), miR-146a (**F**) (*IRAK1* (**G**), *TRAF6* (**H**)), miR-223 (**I**) (*TLR4* (**J**), *IKKα* (**K**), *NLRP3* (**L**)), and miR-125b (**M**) (*MyD88* (**N**)) in the lungs during rabbits infection with *Lagovirus europaeus*—two genotypes (GI.1 (*n* = 10) and GI.2 (*n* = 10))—and controls (*n* = 10). The expression of all the genes is normalized to an endogenous reference (miR-103a for all tested miRs and 18S rRNA for other genes) and presented as a relative fold change to the controls according to the comparative Ct method (2^−ΔΔCt^). The miR and target gene levels were evaluated using real-time PCR. Data were compared with the one-way ANOVA test or the ANOVA Kruskal–Wallis test. The *t*-test, or Mann–Whitney U test, was performed to assess the differences in parameter concentrations. *p*-values below 0.05 were considered statistically significant. Bars indicate the mean ± standard error of the mean (SEM), * *p* < 0.5, ** *p* < 0.01, and *** *p* < 0.001.

In the lung, changes in miR levels were observed only in two of four studies miRs. They were miR-146a and miR-223. No change was detected for the miR-155 (Figure 2A) and its target genes *TAB2* and *p65* (Figure 2C,D), except *MyD88* and *NLRP3* (Figure 2B,E). Our research showed an increase in the mRNA level for *MyD88* (3-fold change in the GI.1 group (*p* = 0.007) and 5-fold change in the GI.2 group (*p* = 0.003); Figure 2B). The highest increase in expression compared to the control was recorded in the *NLRP3* gene (5-fold (*p* < 0.001) for the GI.1 and the GI.2 −5.8-fold (*p* < 0.001) (Figure 2E). 

During *L. europaeus* infection of both genotypes, our studies showed an increase in expression of miR-146a (2.4-fold change for GI.1 vs. control, *p* < 0.001 and 4.5-fold change for GI.2 vs. control, *p* < 0.001; Figure 2F). The increase in miR-146a was associated with a decrease in *IRAK1* and *TRAF6* gene expression. The decrease in the expression level of *IRAK* was similar in both infected groups (GI.1 and GI.2) and amounted to a 1.6-fold reduction for GI.1 group (*p* = 0.03) and a 1.7-fold reduction for GI.2 (*p* = 0.02) compared to the control (Figure 2G). However, during *L. europaeus* GI.1 infection we noted a downregulation of *TRAF6* was a 2-fold reduction (*p* = 0.02) compared to the control, while during *L. europaeus* GI.2 genotype infection a downregulation of *TRAF6* was 2.9-fold reduction (*p* = 0.005 vs. control) (Figure 2H).

Different expressions of miR-223 were observed in the lungs compared to the liver. The expression level of miR-223 was significantly lower in the lungs in both infected groups of rabbits (1.5-fold reduction (*p* = 0.017) for GI.1 and 1.9-fold reduction (*p* = 0.014) for GI.2 (Figure 2I). The downregulation of miR-223 was accompanied by increased expression of all target genes. The expression of *TLR4* for GI.1 and GI.2 group increased 2.7-fold, (*p* < 0.001 vs. control) and 3-fold, (*p* < 0.001 vs. control), respectively (Figure 2J). In the case of the *IKKα* gene, we observed an upregulation by a 3.5-fold change in the GI.1 infected group (p < 0.001 vs. control) and a 3.7-fold change in the GI.2 group (*p* = 0.004 vs. control) (Figure 2K). However, the highest increase in expression compared to the control was recorded in the *NLRP3* inflammasome gene (5-fold (*p* < 0.001) for the GI.1 and 5.8-fold (*p* < 0.001) for the GI.2) (Figure 2L). Similarly to miR-155, our studies showed no change in the expression level of miR-125b (Figure 2M), but with a clearly marked increase in *MyD88* expression (3-fold change in GI.1 group (*p* = 0.007) and 5-fold change in GI.2 (*p* = 0.003); Figure 2N).

#### 2.1.3. Kidney

In the kidneys, our studies showed an increase in the expression of almost all tested miRs during infection with both L. europaeus genotypes except miR-125b. 

**Figure 3:**
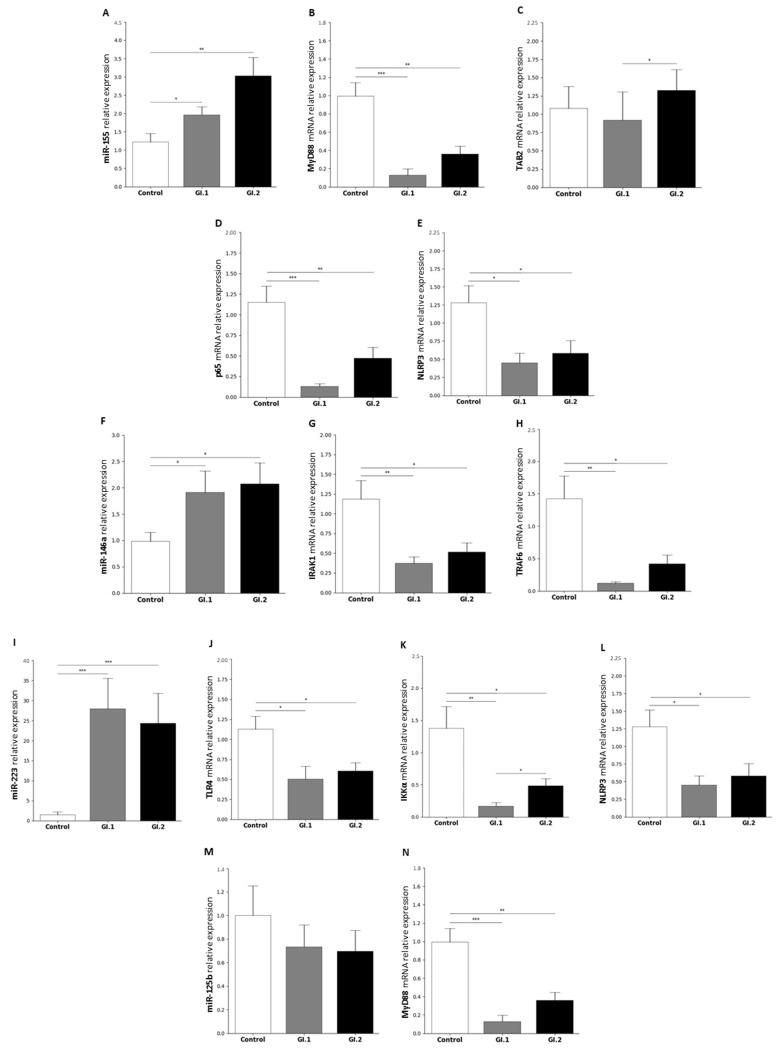
Relative expression of miRs and downstream targets: miR-155 (**A**) (*MyD88* (**B**), *TAB2* (**C**), *p65* (**D**), *NLRP3* (**E**)), miR-146a (**F**) (*IRAK1* (**G**), *TRAF6* (**H**)), miR-223 (**I**) (*TLR4* (**J**), *IKKα* (**K**), *NLRP3* (**L**)), and miR-125b (**M**) (*MyD88* (**N**)) in the kidney during rabbits infection with *Lagovirus europaeus*—two genotypes (GI.1 (*n* = 10) and GI.2 (*n* = 10))—and controls (*n* = 10). The expression of all genes is normalized to an endogenous reference (miR-103a for all tested miRs and 18S rRNA for other genes) and presented as a relative fold change to the controls according to the comparative Ct method (2^−ΔΔCt^). The miR and target gene levels were evaluated using real-time PCR. Data were compared with the one-way ANOVA test or the ANOVA Kruskal–Wallis test. The *t*-test, or Mann–Whitney U test, was performed to assess the differences in parameter concentrations. *p*-values below 0.05 were considered statistically significant. Bars indicate the mean ± standard error of the mean (SEM), * *p* < 0.5, ** *p* < 0.01, and *** *p* < 0.001.

The expression of miR-155 was significantly different in the infected rabbits compared to the control rabbits during infection with *L. europaeus* (1.6-fold change, *p* = 0.03 for GI.1 and 2.5-fold change, *p* = 0.004 for GI.2) (Figure 3A). 

Overexpression of miR-155 was associated with decreased expression levels of *MyD88* (7.7-fold reduction for GI.1, *p* < 0.001 vs. control and 2.7-fold reduction for GI.2, *p* = 0.001 vs. control; Figure 3B) and *p65* (8.9-fold reduction for GI.1, *p* < 0.001 vs. control and 2.4-fold reduction for GI.2, *p* = 0.009 vs. control; Figure 3D) in both infected groups. Whereas no statistically significant change was observed in the expression level of the *TAB2* gene (Figure 3C). The studies showed only a decrease in the *TAB2* gene (1.4-fold reduction, *p* = 0.038) between the GI.1 and GI.2 infected groups (Figure 3C). In the case of the *NLRP3* inflammasome gene, a decreasing expression was observed (2.6-fold reduction during *L. europaeus*/GI.1 (*p* = 0.02) and 1.7-fold reduction during GI.2 (*p* = 0.03) infection compared to the control; Figure 3E). 

Upregulation of miR-146a (1.9-fold change for GI.1, *p* = 0.047 vs. control and 2-fold change for GI.2, *p* = 0.02 vs. control; Figure 3F) in the kidney was associated with downregulation of *IRAK1* and *TRAF6*. In the GI.1 group, the reduction of *IRAK1* was a 3.2-fold reduction, while in GI.2 it was a 2.3-fold reduction (Figure 3G). Compared to the control, the level of *TRAF6* expression decreased during infection of GI.1 (11.5-fold reduction (*p* = 0.002)), while during GI.2, it was only a 3.4-fold reduction (*p* = 0.017) (Figure 3H). *TRAF6* gene expression was also decreased between groups (3.4-fold lower in the GI.1 than in the GI.2 (*p* = 0.045); Figure 3H).

Similarly to the liver, the highest increase in expression among miRs was observed in the case of miR-223. The greatest increase occurred during infection with the *L. europaeus*/GI.1 genotype (18.6-fold change compared to the control (*p* < 0.001)). Followed by GI.2 (16.3-fold change vs. control (*p* < 0.001)) (Figure 3I). 

All target genes for MiR-223 were downregulated. The expression of *TLR4* for the GI.1 and GI.2 group decreased by a 2.8-fold reduction, (*p* < 0.017 vs. control) and 1.8-fold reduction, (*p* < 0.011 vs. control), respectively (Figure 3J). In the case of the *IKKα* gene, we observed an 8.4-fold reduction in the GI.1 group (*p* = 0.001 vs. control) and a 2.8-fold reduction in GI.2 (*p* = 0.02 vs. control) (Figure 3K). Additionally, the expression of *IKKα* was a 2.9-fold reduction in the case of GI.1 infection compared to the GI.2 infected group (*p* = 0.03; Figure 3K). Furthermore, the expression of *NLRP3* was downregulated (2.6-fold reduction, *p* = 0.021 and 1.7-fold reduction, *p* = 0.028, during GI.1 and GI.2 infection) compared to healthy rabbits (Figure 3L). Our study showed no change in the expression level of miR-125b in both infected groups (Figure 3M). However, the expression level of the *MyD88* gene decreased in both groups (7.7-fold reduction during GI.1 (*p* = 0.02) and 2.7-fold reduction during GI.2 (*p* = 0.03) infection compared to the control) (Figure 3N).

#### 2.1.4. Spleen

**Figure 4:**
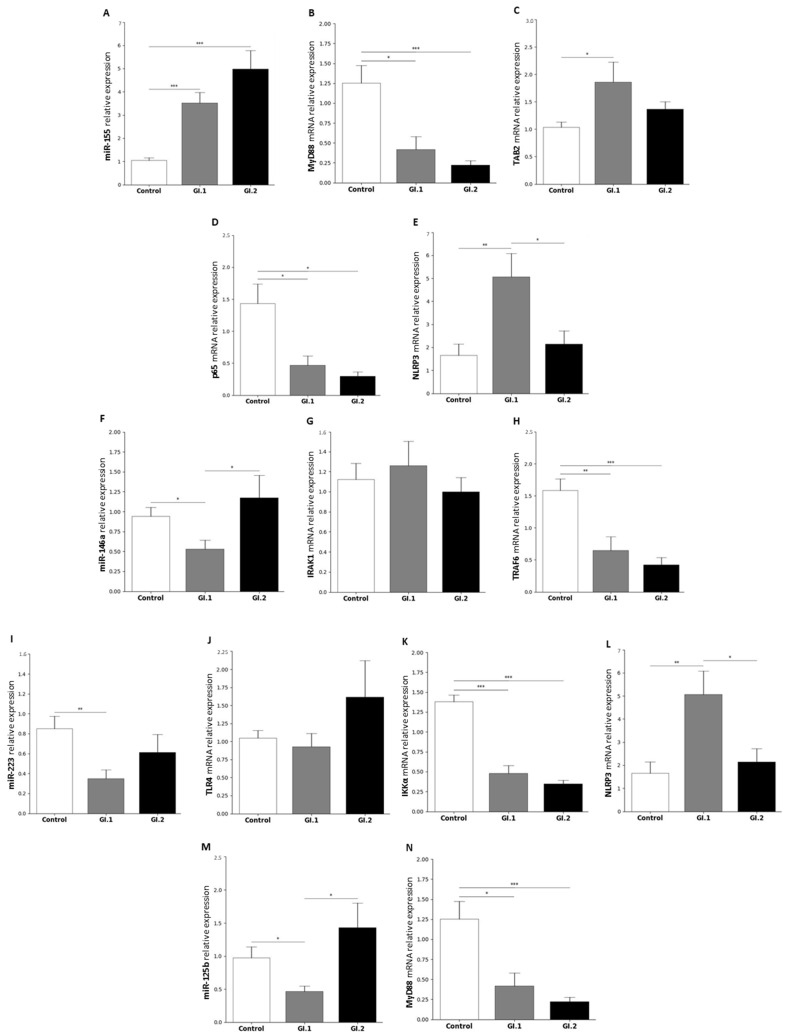
Relative expression of miRs and downstream targets: miR-155 (**A**) (*MyD88* (**B**), *TAB2* (**C**), *p65* (**D**), *NLRP3* (**E**)), miR-146a (**F**) (*IRAK1* (**G**), *TRAF6* (**H**)), miR-223 (**I**) (*TLR4* (**J**), *IKKα* (**K**), *NLRP3* (**L**)), and miR-125b (**M**) (*MyD88* (**N**)) in the spleen during rabbits infection with *Lagovirus europaeus*—two genotypes (GI.1 (*n* = 10) and GI.2 (*n* = 10))—and controls (*n* = 10). The expressions of all genes are normalized to an endogenous reference (miR-103a for all tested miRs and 18S rRNA for other genes) and presented as a relative fold change to the controls according to the comparative Ct method (2^−ΔΔCt^). The miR and target gene levels were evaluated using real-time PCR. Data were compared with the one-way ANOVA test or the ANOVA Kruskal–Wallis test. The *t*-test, or Mann–Whitney U test, was performed to assess the differences in parameter concentrations. *p*-values below 0.05 were considered statistically significant. Bars indicate the mean ± standard error of the mean (SEM), * *p* < 0.5, ** *p* < 0.01, and *** *p* < 0.001.

Our studies show that only miR-155 is upregulated in the spleen during infection with both *L. europaeus* genotypes. MiR-155 was upregulated by a 3.3-fold change (*p* < 0.001) for the GI.1 group, while for GI.2, there was a 4.7-fold change (*p* < 0.001) compared to the control (Figure 4A). As in the kidney, the increase in miR-155 was associated with a decrease in its target genes, *MyD88* and *p65*. The expression levels of *MyD88* for the GI.1 and GI.2 groups decreased by a 3-fold reduction (*p* = 0.026 vs. control) and 5.6-fold reduction (*p* < 0.001 vs. control), respectively (Figure 4B). Expression of the *p65* gene was a 3-fold reduction (*p* = 0.038) in the case of infection with the *L. europaeus*/GI.1 genotype and 4.8-fold lower (*p* = 0.01) in GI.2 (Figure 4D). In the spleen, the change in *TAB2* gene expression was observed only during infection with the GI.1 genotype (1.8-fold change (*p* = 0.04) compared to the control) (Figure 4C).

The remaining miRs were expressed only during infection with the GI.1 genotype, and all of them showed reduced expression. The expression level of miR-146a was downregulated by a 1.8-fold reduction (*p* = 0.017; Figure 4F). However, no change in the expression level of the target gene *IRAK1* was observed in the GI.1 group compared to the control group (Figure 4G). Moreover, in the second group, GI.2, *IRAK1* also showed no altered expression (Figure 4G). However, interestingly, our studies showed a decrease in *TRAF6* expression in both infected groups (2.5-fold reduction for GI.1, *p* = 0.0046 and 3.7-fold reduction for GI.2, *p* < 0.01) compared to the healthy rabbits (Figure 4H).

The expression level of miR-223 was significantly lower for the GI.1 genotype (2.5-fold reduction compared to the control; (*p* = 0.005); Figure 4I). No changes in *TLR4* gene expression were observed in both infected groups (Figure 4J). Expression of *IKKα* was reduced in both *L. europaeus* infected groups, with a 2.8-fold reduction for GI.1 (*p* < 0.001 vs. control) and a 3.9-fold reduction for GI.2 (*p* < 0.001 vs. control) (Figure 4K). At the same time, the reduction miR-223 was associated with a 3-fold change in *NLRP3* gene expression level (*p* = 0.006; Figure 4L). Additionally, our studies showed a statistically significant difference in *NLPR3* expression between the infected groups (2.3-fold higher in the GI.1 group compared to the GI.2 group (*p* = 0.02); Figure 4L). MiR-125b expression was a 2-fold reduction (*p* = 0.13) only in the group GI.1 compared to the control, and 3-fold lower for the GI.1 group compared to GI.2 (*p* = 0.02; Figure 4M). Additionally, the expression level of the *MyD88* gene decreased in both groups (3-fold reduction for GI.1 (*p* = 0.02) and 5.6-fold reduction for GI.2 (*p* = 0.03) compared to the control) (Figure 4N).

### 2.2. Biomarkers of Inflammation in the Liver, Lung, Kidney, and Spleen during Lagovirus europaeus/GI.1 and GI.2 Genotype Infection in Rabbits

**Figure 5:**
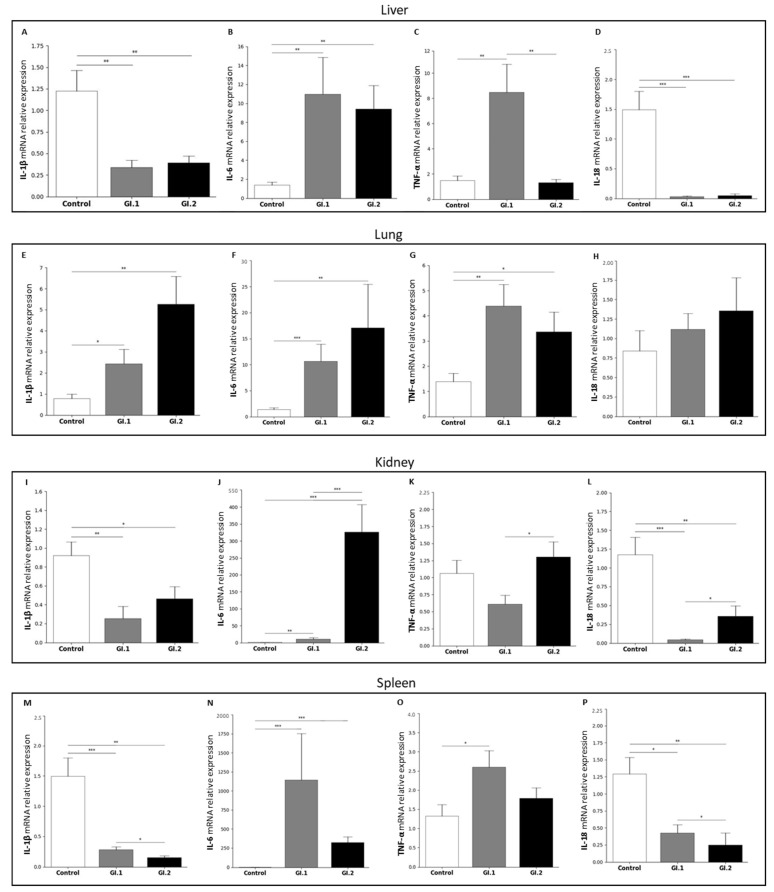
Expression biomarkers of inflammation: IL-1β (**A**,**E**,**I**,**M**), IL-6 (**B**,**F**,**J**,**N**), TNF-α (**C**,**G**,**K**,**O**) and IL-18 (**D**,**H**,**L**,**P**) in the liver (**A**–**D**), lung (**E**–**H**), kidney (**I**–**L**) and spleen (**M**–**P**) of controls rabbits (*n* = 10), and *L. europaeus*/GI.1 (*n* = 10) and GI.2 (*n* = 10) genotype infections. The expressions of all genes are normalized to an endogenous reference (18S rRNA) and presented as a relative fold change to the controls according to the comparative Ct method (2^−ΔΔCt^). The target gene levels were evaluated using real-time PCR. Data were compared with the one-way ANOVA test or the ANOVA Kruskal–Wallis test. The *t*-test, or Mann–Whitney U test, was performed to assess the differences in parameter concentrations. *p*-values below 0.05 were considered statistically significant. Bars indicate the mean ± standard error of the mean (SEM), * *p* < 0.5, ** *p* < 0.01, and *** *p* < 0.001.

**Figure 6:**
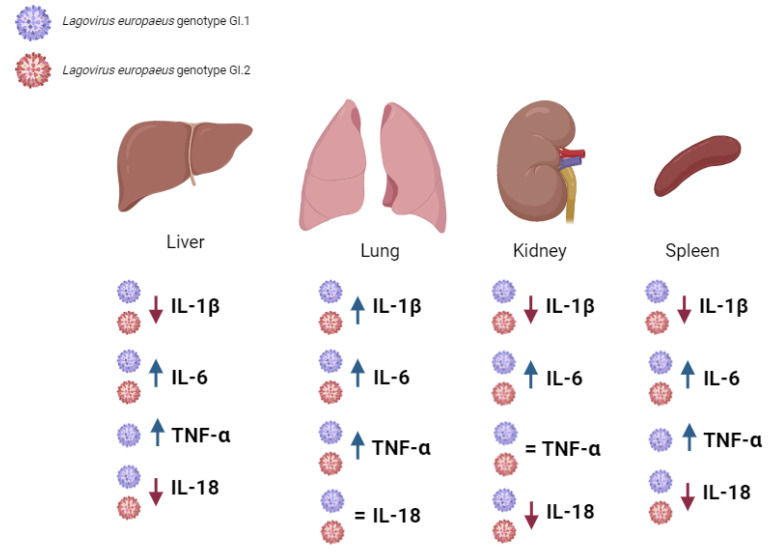
Biomarkers of inflammation during *Lagovirus europaeus*/GI.1 and GI.2 genotype infection in rabbits.

The study aimed to determine the relative level of mRNA expression of selected biomarkers of inflammation, *IL-1β*, *IL-6*, *TNF-α*, and *IL-18* (proinflammatory cytokines), involved in the acute phase response (rapid, non-specific immune and inflammatory response, including systemic metabolic-physiological changes, affecting tissues and organs) (Figure 5 and Figure 6, Table 1).

In the liver the relative level of mRNA expression of *IL-1β* was decreased during infection with *L. europaeus* of both genotypes (3.6-fold reduction for genotype GI.1 (*p* = 0.002) and 3.1-fold reduction for GI.2 (*p* = 0.004)) compared to the control group (Figure 5A). In the case of *IL-6* (the most important mediator of the inflammation, stimulating the synthesis of acute phase protein), we observed its increase after infection with *L. europaeus* of both genotypes (8-fold change, *p* = 0.003 for GI.1 vs. control and 6.8-fold change, *p* = 0.004 for GI.2 vs. control; Figure 5B). It should be emphasized that this cytokine was more strongly expressed in the group of rabbits infected with the *L. europaeus*/GI.1 genotype. In our studies, we also observed an increased level of the main mediator of tissue damage, *TNF-α*, during infection, but only in the GI.1 group (5.7-fold change, *p* = 0.006 vs. control; Figure 5C). Additionally, we observed 6.4-fold higher *TNF-α* expression during infection with the GI.1 genotype compared to the GI.2 infected group (*p* = 0.04; Figure 5C). The expression level of the *IL-18* gene was the lowest in the liver, from all examined tissues, where downregulation was a 46.8-fold reduction (*p* < 0.001) and 31-fold reduction (*p* < 0.001) for GI.1 and GI.2 (Figure 5D).

In the lungs, our studies showed an increase in almost all tested cytokines except *IL-18*, whose expression was unchanged (Figure 5H). The greatest changes in expression levels in the lung were observed in the case of *IL-6*. Compared to the control, the *IL-6* mRNA relative expression was 7.7-fold enhanced for GI.1 (*p* < 0.001) and 12.3-fold enhanced for GI.2 (*p* = 0.002) (Figure 5F). During infection with the *L. europaeus*/GI.1 genotype, we noted a similar increase in the expression level of *IL-1β* and *TNF-α*, which was a 3.1-fold change (*p* = 0.045 for *IL-1β* (Figure 5E) and *p* = 0.004 for *TNF-α* (Figure 5G)). Whereas during the *L. europaeus*/GI.2 infection, the level of *IL-1β* was enhanced by a 6.7-fold change (*p* = 0.045 vs. control; Figure 5E) and a 2.4-fold change for *TNF-α* (*p* = 0.003 vs. control; Figure 5F). 

Similar to the liver, we observed a significant decrease in *IL-1β* and *IL-18* levels but also increased levels of *IL-6* in the kidney and the spleen. In the kidney, expression of *IL-1β* was decreased by a 3.6-fold reduction (*p* = 0.003 vs. control; Figure 5I) in the GI.1 group and a 2-fold reduction for *IL-1β* in GI.2 (*p* = 0.04; Figure 5I). The expression of *IL-6* was significantly increased in the rabbits infected with the *L. europaeus*/GI.1 and GI.2 genotypes, with an 8.5-fold change (*p* = 0.004) and 261-fold change (*p* < 0.001), respectively (Figure 5J). We also noted a difference in *IL-6* expression levels between infected groups. During *L. europaeus* GI.1 infection, the level of *IL-6* expression was 30-fold lower compared to the GI.2 group (*p* < 0.001; Figure 5J). Our study showed no change in *TNF-α* expression in the kidneys in both infected groups compared to the control group (Figure 5K). However, we observed a statistically significant change in the *TNF-α* expression level in both infected groups (2.1-fold lower in the GI.1 group (*p* = 0.003) vs. GI.2; Figure 5K). In the kidney In the GI.1 group, expression of *IL-18* was decreased by a 27.5-fold reduction (*p* = 0.01 vs. control; Figure 5L). However, for the GI.2 genotype, we noted a 3.3-fold reduction in *IL-18* (*p* = 0.004; Figure 5L) compared to the control. Our studies showed a difference in expression levels of *IL-18* between rabbits infected with *L. europaeus* for both genotypes.Comparing GI.1 to the GI.2 group, the level of *IL-18* was downregulated (8.3-fold reduction (*p* = 0.03); Figure 5L). 

In the spleen, the relative expression of *IL-1β* mRNA was a 5.5-fold reduction for GI.1 (*p* = 0.04) and a 1.9-fold reduction for GI.2 (*p* = 0.004) compared to the healthy tissues (Figure 5M). Additionally, we observed 5.2-fold higher *IL-1β* expression during infection with the GI.1 genotype compared to the GI.2 infected group (*p* = 0.04; Figure 5M). Our studies have shown that during infection with both *L. europaeus* genotypes, the level of *IL-6* increases most in the spleen. During infection with the GI.1 genotype, the expression level increased by a 997-fold change (*p* < 0.001) compared to the control group, while for the GI.2 genotype, the increase was a 282-fold change (*p* < 0.001) (Figure 5N). In the case of *TNF-α*, our studies show an increase in the relative expression of mRNA only during infection with the GI.1 genotype (1.9-fold change, *p* = 0.02 vs. control; Figure 5O). Similarly to other tissue, in the spleen, we observed a decrease in expression levels of *IL-18* during *L. europaeus* infection with both genotypes. Compared to the control, in the GI.1 group, the decrease was a 3-fold reduction (*p* = 0.045), while in the GI.2 group, it was a 5.1-fold reduction (*p* = 0.004) (Figure 5P). Similar to *IL-1β*, our studies showed a change in *IL-18* expression levels between infected groups (1.7-fold change for GI.1, *p* = 0.038 vs. GI.2; Figure 5P). 

### 2.3. Clinical Signs of Disease and Post-Mortem Analysis

Animals infected with both *L. europaeus* genotypes—GI.1 and GI.2—showed clinical signs consistent with RHD (apathy, dyspnea, body temperature > 41 °C, anorexia, and neurological symptoms). Post-mortem findings of infected rabbits showed characteristic anatomopathological organ changes during RHD. Two rabbits after the *L. europaeus*/GI.2 infection died asymptomatically. Mortality after infection with *L. europaeus* in both genotypes was 90% at 60 hpi. The *L. europaeus*/GI.2 genotype was more virulent, causing 90% mortality in rabbits within 32 hpi and a fulminant course of the disease. The disease ranged from per-acute to acute in animals infected with this *L. europaeus* genotype. Whereas, after *L. europaeus*/GI.1 infection, the mortality rate was −10% at 32 hpi, 40% at 36 hpi, and 40% between 56 and 60 hpi.

## 3. Discussion

So far, apart from our studies [16,23,24,25,53], there is a lack of information on the molecular signatures of regulatory interactions between miRs and biological processes involved in *L. europaeus* infection/RHD pathogenesis. We examined miRs and target genes involved in regulating the innate immune and inflammatory response. To investigate the immune and inflammatory response to the viral stimulus in the examined tissues, we examined biomarkers of the inflammation *IL-1β*, *IL-6*, *TNF-α*, and *IL-18* involved in acute phase response [54,55,56,57,58].

The immune and inflammatory response is the coordinated activation of many cells, substances, and signaling pathways that regulate the levels of inflammatory mediators in tissue-resident cells and inflammatory cells recruited from the blood. Although inflammatory response processes depend on the exact nature of the initial stimulus and its location in the body, they all share a common mechanism: cell surface receptors recognize harmful stimuli; inflammatory pathways are activated; inflammatory biomarkers are released; and inflammatory cells are recruited [59].

For the first time, we examined inflammatory biomarkers (in liver, lungs, kidneys, and spleen) in rabbits infected with *L. europaeus*—two genotypes: GI.1 and GI.2. We examined the expression level of cytokines *IL-1β*, *IL-6*, *TNF-α*, and *IL-18* in each post-mortem organ. Our research shows that the expression profile of biomarkers of inflammation in rabbits infected with *L. europaeus* is similar for GI.1 and GI.2 genotypes in terms of the nature of changes (increase/decrease/no changes in expression) but is different for individual tissues.

The inflammatory biomarker profile in the liver of infected rabbits was characterized by a decrease in *IL-1β* expression at very similar levels after infection with both genotypes (3.6-fold reduction and 3.1-fold reduction for GI.1 and GI.2, respectively). An increase in *IL-6* mRNA expression for both genotypes was slightly higher in the case of the GI.1 (8-fold change) genotype. The rise in *TNF-α* expression after *L. europaeus*/GI.1 infection was a 5.7-fold change and 6.4-fold higher *TNF-α* expression in GI.1 compared to the GI.2 infection group. This fact suggests the development of an inflammatory response and liver damage. We also recorded a drastic reduction in the level of *IL-18* mRNA expression after *L. europaeus* infection (both genotypes), with a stronger reduction in the GI.1 infection group (46.8-fold reduction).

In turn, the inflammatory biomarker profile in the lungs of infected rabbits was characterized by an increase in the expression of three acute-phase cytokines (*IL-1β*, *IL-6*, and *TNF-α*). After infection with the *L. europaeus*/GI.2 genotype, *IL-1β* expression in the lungs increased by 6.7-fold, and 3.1-fold changes for GI.1. We recorded a similar situation for *IL-6* (12.3-fold change, GI.2; 7.7-fold change, GI.1). Moreover, an increase in the expression of *TNF-α* after infection with the GI.1 and GI.2 genotypes (3.1-fold change, and 2.4, for GI.1 and GI.2) at a similar level indicates a similar degree of lung damage (slightly more significant for GI.1), perhaps due to the more extended survival period of the animals. In the lung, *IL-18* expression remained unchanged in response to *L. europaeus* infection (both genotypes).

The profile of Inflammatory biomarkers In the kidneys Is characterized by a decrease in *IL-1β* mRNA expression with both genotypes. There was a critical increase in the level of *IL-6* expression in response to infection with *L. europaeus*, genotype GI.2 (261-fold change), and much smaller in the GI.1 group (8.5-fold change). In the kidney, unlike all other tissues examined, *TNF-α* expression remained unchanged in response to infection. Similarly to the liver, infection with *L. europaeus*/GI.1 and GI.2 drastically reduced the expression level of *IL-18* in the kidneys by a 27.5-fold and 3.3-fold reduction.

The profile of Inflammatory biomarkers In the spleen regarding the nature of changes (increase/decrease expression) was very similar to the liver. There was a 5.5-fold reduction in *IL-1β* for GI.1 and a 1.9-fold reduction for GI.2. The highest increase in *IL-6* mRNA expression (997-fold change) was observed in rabbits infected with the GI.1 and with GI.2 genotype (282-fold change). Increased *TNF-α* expression was observed after *L. europaeus*/GI.1 infection at the level of a 1.9-fold change. We also recorded a reduction in the level of *IL-18* mRNA expression after *L. europaeus* infection (both genotypes), with a stronger expression in the GI.2 infection group (5.1-fold reduction) and GI.1 (3-fold reduction).

Our research shows that infection of rabbits with *L. europaeus*/GI.1 and GI.2 genotypes causes an increase in the expression of two critical acute phase cytokines—*IL-6* in all examined tissues (liver, lungs, kidneys, and spleen) and *TNF-α* (in the liver, lungs, and spleen). Cytokine *IL-1β* was highly expressed only in the lungs after *L. europaeus* infection—both genotypes. *IL-6* production following *L. europaeus* infection/GI.1 and GI.2 was most dramatic in the spleen (for GI.1 and GI.2, respectively), kidney (GI.2 and GI.1), lung (GI.2 and GI.1) and liver (GI.1 and GI.2). Increases in *TNF-α* expression were most significant in the liver (for GI.1 only), lung (GI.1 and GI.2), and spleen (GI.1 only). These facts indicate a strong and rapid involvement of the local innate immune and inflammatory response in *L. europaeus* infection for both genotypes (GI.1 and GI.2) and in the pathogenesis of RHD. Dramatic levels of cytokines in tissues may drive the pathogenesis of RHD, which may lead to hypercoagulability, formation of microthrombi (as observed post-mortem), acute liver failure, multi-organ failure, coagulopathy with hemorrhage and ultimately, death of animals. The marked upregulation and overexpression of cytokines-*IL-6* and *TNF-α* in the examined organs and *IL-1β* (only in the lungs) is probably for the resulting per-acute/acute (in the case of *L. europaeus*/GI.2 infection) and acute (in the case of *L. europaeus*/GI.1 infection) form of the disease and mortality of animals.

IL-6 is a promising biomarker of the early phase of inflammation because it has a longer half-life than other cytokines (e.g., IL-1β), and its concentration in blood and tissues may increase several thousand times within the first 2–3 h after the initiation of inflammatory processes, reaching critical values in fatal states [60], which we also recorded in our research. Monocytes, macrophages, and neutrophils produce it. Its primary role is in the regulation of anti-infective immune response and inflammation. Moderate and acute increases in IL-6 levels contribute to hepatocyte regeneration. However, persistently elevated levels of IL-6 lead to apoptosis of hepatocytes, other cells, damage to the vascular endothelium, and activation of coagulation [61]. 

In turn, TNF-α is one of the central cytokines of the inflammatory response with proinflammatory and anti-infective effects [62,63,64]. The biological effects of TNF-α depend on the intensity of secretion of this cytokine. Rapid secretion of large amounts of TNF-α leads to shock, acute respiratory failure/lung damage, hepatocyte apoptosis, adrenal hemorrhage, and DIC [64]. In turn, chronic secretion of small amounts of this cytokine causes liver and spleen enlargement. TNF-α blockade in an animal model with induced ALF led to attenuated hepatocyte damage and increased regeneration [64]. Changes in the level of TNF-α can predict COVID-19 progression, lung damage, and disease severity. Using TNF-α inhibitors or blockers to treat COVID-129 can prevent mortality in severe COVID-19 patients [63]. O’Toole et al. [42] described for the first time the profile of acute phase cytokines (IL-6, IL-1β, TNF-α) after administration to rabbits per os inoculum (in the form of liver homogenate containing the *L. europaeus*/GI.2 genotype). Similarly, they found increased expression of *IL-6* in the liver and spleen (at 36 and 48 hpi) and increased expression of *TNF-α* in the liver and spleen (from 12 h to 144 hpi). Similar to our study, Yu et al. [43] showed that after infection of rabbits with the *L. europaeus*/GI.2 genotype, there is an increase in *IL-6* expression in the spleen. Other researchers [37,39,41] noticed increased expression of *IL-6* and *TNF-α* during *L. europaeus* infection, GI.1 genotype, in serum and peripheral blood leukocytes. Marques et al. [41] observed an increase in the expression of *IL-6* (from 24 h) and *TNF-α* (48 h) in the serum, and similar to Semeryjan et al. [39], an increase in *TNF-α* (1 and 2 dpi). Trzeciak-Ryczek et al. [37] showed increased *IL-6* and *TNF-α* (36 and 48 hpi) expression in peripheral blood leukocytes.

Our study only found increased *IL-1β* mRNA expression in the lungs (GI.2 and GI.1, respectively). IL-1β is secreted mainly by monocytes and macrophages from various tissues by microbial products that activate PRR receptors and other cytokines. IL-1, systemically and at the site of inflammation, causes biological effects that facilitate the development of the inflammatory reaction, among others, and induces increased production of neutrophils and monocytes. It plays a crucial role in the acute phase of inflammation by inducing IL-6 [55]. The short half-life of IL-1β may limit its use in diagnosing the inflammatory process, which may have been the case in our studies [60]. Our studies’ results differ from those obtained by O’Toole et al. [42] and Yu [43] after the infection of rabbits with the *L. europaeus*/GI.2 genotype. The first team [42] showed an increase in *IL-1β* expression in the liver (36 hpi and 48 hpi) and spleen (36, 48, 144 hpi). Yu et al. [43] showed increased *IL-1β* expression in the spleen. The same results after infection with *L. europaeus*—genotype GI.1—was reported by Marques et al. [41], showing an increase in *IL-1* (up to 18 hpi) and Trzeciak-Ryczek et al. [38], showing a decrease in *IL-1β* (8 hpi, and 24 and 28 hpi) in peripheral blood leukocytes.

Our study also found dramatically reduced levels of *IL-18* mRNA expression in the liver, kidney, and spleen. In the lungs, *IL-18* expression remained unchanged. The literature data indicate that IL-18 strategically generates the immune response against viral infections and is usually proinflammatory [56,57]. Macrophages produce IL-18 at the very early stages of viral infection and induce the production of IL-6 and IFN-γ. As a rule [58], an increase in the level of IL-18 in plasma is observed during viral infections. Infections with EBV, HIV, chronic HB and HC infections, and acute dengue infections have been evident of this fact. Moreover, circulating IL-18 and ferritin levels may strongly correlate with dengue disease severity and can be considered a tool for predicting disease progression [58]. Our results are difficult to interpret unambiguously at this research stage because, as Slaats et al. [58] point out, distinct inflammatory programs in *IL-18*-mediated viral infection require investigation. Our study results are consistent with Trzeciak-Ryczek et al. [37], who recorded decreased *IL-18* expression (8 hpi and between 24 and 36 hpi) in peripheral blood leukocytes during *L. europaeus*/GI.1 infection. Moreover, the study by these authors [37] showed a negative correlation between reduced *IL-18* expression and the survival time of infected rabbits (36 h).

Our studies confirm that in *L. europaeus* infection/GI.1 and GI.2 genotypes, miRs are crucial in regulating innate immune and inflammatory responses. MiRs, which regulate inflammation, may have pro- and anti-inflammatory effects [46,47,49,50]. MiRs regulate complex gene networks, leading to the observation of opposite phenotypes for the same miR. The levels of miRs (miR-155, miR-146a, miR-223, miR-125b) induced after a viral stimulus can be different and usually lead to placing the cell in a specific state. Induced miRs will be targeted to different pro- or anti-inflammatory functions during the inflammatory response, depending on their target genes [46,47,49,50]. 

Our studies indicate that in the liver, miR-155 has both pro-inflammatory and anti-inflammatory effects, while miR-146a, miR-223, and miR-125b have anti-inflammatory effects by regulating its target genes that are critical in the TLR4-MyD88, NF-ĸβ, and NLRP3 inflammasome pathway [46]. The dramatic increase in liver miR expression was miR-223 after infection with *L. europaeus*/GI.1 and GI.2. A similar regulation of miR-223 has been observed in studies on EBV [65], SARS-CoV-1 [66,67], HB virus [68], HIV [68], in liver diseases [69,70], and lung diseases [71]. After *L. europaeus* infection, miR-223 significantly increased and dramatically decreased the expression of the *IKKα* gene at various levels, which was also observed in the kidney. The correlation confirmed the inhibitory effect of miR-223 on the *IKKα* gene in both genotypes. The literature reports [46] that miR-223 responds to *TLR4* by binding its promoter to NF-κB. This induction of miR-223 acts as a negative feedback loop because miR-223 targets the *IKKα* gene, thereby attenuating the TLR/NF-κB signaling pathway. Additionally, miR-223 also targets the *NLRP3* gene, reducing caspase-1 activation and subsequent IL-1β processing [46,47]. Our study indicates that miR-155, a well-described regulator of inflammation [46,47], has anti-inflammatory and pro-inflammatory effects in the liver in *L. europaeus*/RHD infection. On the one hand, miR-155 dramatically inhibited *MyD88* (an innate immunity gene necessary for the activation of immune cells via TLRs) and the *p65* subunit of the main transcription factor NF-ĸβ, on which the further fate of the immune and inflammatory response depends. On the other hand, miR-155 exhibited pro-inflammatory effects by upregulating the *NLRP3* inflammasome, a critical component of the innate immune response mediating the severity of inflammation through the secretion of pro-inflammatory cytokines, IL-1β and IL-18, in response to viral infection and cell damage [72,73]. Moreover, the increase in miR-125b expression in the liver inhibited the expression of the *MyD88* gene, which indicates its anti-inflammatory potential. According to one theory, miR-125b confers its anti-inflammatory potential probably by targeting TRAF6-mediated NF-ĸB signaling, thereby regulating inflammatory gene expression [46]. It is important to emphasize that researchers observed such regulation of miR-125 in the liver and spleen. In turn, overexpression of miR-146a in the liver was manifested by a regulatory effect on the critical immune gene *TRAF6* (a gene in the NF-ĸβ pathway that activates IKK in response to inflammatory cytokines) [74], causing its decrease. We also recorded increased expression of the *IRAK1* gene (which plays a crucial role in the innate immune response to viruses by inducing acute inflammation) [74]. Further research on these miRs focuses on explaining why, despite the identical overexpression of miR-146a in both GI.1 and GI.2 groups, the decrease in *TRAF6* in the GI.2 group is so significant compared to the GI.1 group.

Our study showed that in the lung as opposed to the liver, altered miRs expression is observed only in the case of miR-146a and miR-223. Our studies indicate that miR-146a and miR-223 have anti-inflammatory effects in the lung. The expression of miR-146a increased in the leach after viral infection, while miR-223 decreased. Limited data on the regulation of miR-223 in different tissue microenvironments indicate that miR-223 may, with varying biological effects, regulate immune target genes and inhibit the synthesis of inflammatory mediators or impede inflammatory signaling pathways, thereby protecting the body from inflammatory damage, which may also take place in the lungs [71]. In the lungs, we showed a decrease in miR-223 expression (similar in both groups), accompanied by an increase in *TLR4*, *IKKα*, and *NLRP3* expression. Researchers have not previously observed such regulation in any of the examined tissues. The correlations performed show a robust negative correlation between miR-223 and its target genes, allowing us to conclude the proposed anti-inflammatory effect of miR-223 in the lungs. However, the observed decrease in miR-223 expression may increase the desired state due to the lack of a mechanism that inhibits target genes involved in the NF-kB and NLRP3 pathways. The limited data on miR-223, NF-ĸβ (p65 subunits), and NLRP3 signaling are inconsistent. In acute lung injury, miR-223 may regulate the inflammatory response during ALI/ARDS [71]. Moreover, in vitro experiments have shown that reduced miR-223 expression reduces the NLRP3 inflammasome and inhibits the TLR4/NF-κB signaling pathway, leading to exacerbation of lung injury [71]. Other studies have shown that during SARS-CoV-2 pneumonia [66], inhibition of miRNA-223-3p increases mRNA levels of pro-inflammatory cytokines and the NLRP3 inflammasome, suggesting that during lung infection, miRNA-223 may contribute to limiting excessive inflammatory response. We propose this explanation for miR-223 lung regulation in *L. europaeus* infection, GI.1 and GI.2 genotypes. In the lungs, miR-125b and miR-155 expression did not change, but we noted an increase in *MyD88* gene expression in the GI.1 and GI.2 groups. However, this increase may be due to increased *TLR4* expression, which may increase transcriptional initiation rates, thereby increasing *MyD88* expression [75]. Our study showed that miR-146a suppresses the expression of critical immune genes *IRAK1* and *TRAF6*, supporting its anti-inflammatory effects. The correlation confirms the regulatory impact of miR-146a on target genes in our research model analyses.

Our studies indicate that miR-155, miR-146a, and miR-223 have anti-inflammatory effects in the kidney by downregulating their target genes, which are critical in the NF-ĸB pathway and may influence its silencing. The expression of the miRs mentioned above increased in response to *L. europaeus* infection with both genotypes. However, no statistically significant changes in miR-125b were observed. The most significant increase in expression in this tissue was for miR-223 (for GI.1 and GI.2). The rise in miR-223 inhibited *TLR4* gene expression at a very similar level after *L. europaeus* infection with both genotypes. MiR-223 also had an inhibitory effect on the *IKKα* gene in case of infection with two genotypes. Additionally, researchers manifested the excessive expression of miR-223 (indicating its anti-inflammatory role in infection with this virus) by inhibiting the expression of the *NLRP3* inflammasome gene. In turn, miR-155, as the primary regulator of inflammation, influenced the inhibition of the *MyD88* gene and caused a decrease in the expression of the *p65* subunit of the main transcription factor NF-ĸβ. In the latter case, the literature data indicate down-regulation of the p65-NF-ĸβ-subunit by miR, which may consequently suppress pro-inflammatory pathways [76]. Researchers have shown that the *TAB* gene, necessary for activating the NF-kB pathway, is a target gene of miR-155. Overexpression of miR-155 significantly decreases the *TAB2* transcript [77]. Our studies did not demonstrate the regulatory effect of miR-155 on the *TAB2* gene, which once again—similarly in the lungs—highlights the different behavior of this gene than previously described [77,78]. This event may suggest that other mechanisms of *TAB2* induction require further investigation. However, miR-146a affected the critical immune genes *IRAK1* and *TRAF6*, causing their decline after *L. europaeus* infection. This effect was also observed in the lungs.

In the spleen, changes in the expression levels of the tested MiRs were most diverse. Our result can be explained by the recent research by Yu et al. [43] regarding transcriptional profiles revealing inflammatory disorders conferred by GI.2 genotype/RHDV2 infection. Infection of rabbits with GI.2 in the spleen elicits a significant upregulation in the expression of numerous genes associated with disease, signal transduction, cellular processes, and cytokine signaling categories. Notably, there was an upregulation in the expression of cytokines and chemokines involved in inflammation. These findings suggested that viral infection could disrupt the cytokine network within the spleen and lead to inflammatory disorders. Our research indicates that miR-155 and miR-223 exert anti-inflammatory effects in the spleen. However, determining the nature of miR-146a and miR-125b remains challenging, and their roles are marked with a question mark in the proposed pathways. Expression of miR-155 increased in response to infection, while miR-146a, miR-223, and miR-125b decreased. Our study showed that miR-155 suppresses the expression of two critical immune genes, *MyD88* and *p65*, both in the GI.1 and GI.2 groups, which supports its anti-inflammatory effect, confirmed by correlation analysis. This fact proves that the action of this part of the pathway in the spleen is very similar to that of the liver. The rise in miR-155 gene expression manifested the increase in *TAB2* expression (only in the GI.1 group). However, correlation analysis did not support this effect. The rise in *TAB2* expression in the spleen is similar to its increase in the liver. Similarly to the liver, it does not correlate with miR-155 levels. Further research is needed to identify the factor that increases *TAB2* expression in the GI.1 and GI.2 genotypes. However, a reduction in miR-125b accompanied the decrease in *MyD88* expression in the spleen (only in the GI.1 group), which is challenging to interpret at this study stage. In the splenic environment, there was no regulatory effect of miR-146a (in both GI.1 and GI.2 groups) on the target genes *IRAK1* and *TRAF6*. The decrease in miR-146a in the spleen is different from other tissues. The reduction in miR-223 expression in the GI.1 group and the decrease in *IKKα* gene expression are difficult to interpret in the splenic microenvironment. Typically, miR-223 negatively correlates with *IKKα* to inhibit inflammation, as confirmed in SARS-CoV-2 infection, hepatitis, and pneumonia [67,69,71]. In turn, the decrease in miR-223 correlated with the increase in the *NLRP3* gene in the GI.1 group, suggesting an anti-inflammatory effect. This effect was also observed in the lungs. We realize that the environment of the spleen, as the central organ of the systemic immune and inflammatory response, is very complex, as confirmed by recent studies by Yu et al. [43]. Given the results of Yu et al. [43], our results seem to confirm the complexity of miRs expression profiles in the spleen microenvironment and their impact on the regulation of innate and inflammatory immune genes in *L. europaeus* infection. Further research in this area is necessary.

### 3.1. Proposed miRs, Target Genes, and Pathways of Innate Immune and Inflammatory Response during Lagovirus europaeus/GI.1 and GI.2 Infection

Our research shows that miRs may regulate three innate immune and inflammatory response pathways in *L. europaeus* infection. However, the result of this regulation may be influenced by the tissue microenvironment, as observed in studies. (1) TLR4-MyD88 signaling pathway: Activation of *MyD88* by *TLR4* results in signal transduction, where MyD88 (protein) activates IRAK, TRAF6, and TAB2, activating the transcription factor NF-κB (in our studies, *p65* subunit). Although we mention proteins here, our research focused on examining the effects of miRs on target genes involved in this pathway. This factor binds to target elements of the NF-κB response in the genome to drive the expression of genes encoding cytokines [46]. (2) NF-ĸB signaling pathway (p65): The phosphorylation of IKK leads to the release of NF-κB dimers. Phosphorylated NF-κB binds to NF-κB DNA response elements and induces the transcription of target genes [46]. (3) NRLP3 inflammasome pathway: NF-κB can activate the NLRP3 inflammasome, which induces IL-1β and IL-18, thereby inducing an inflammatory response [79].

**Figure 7:**
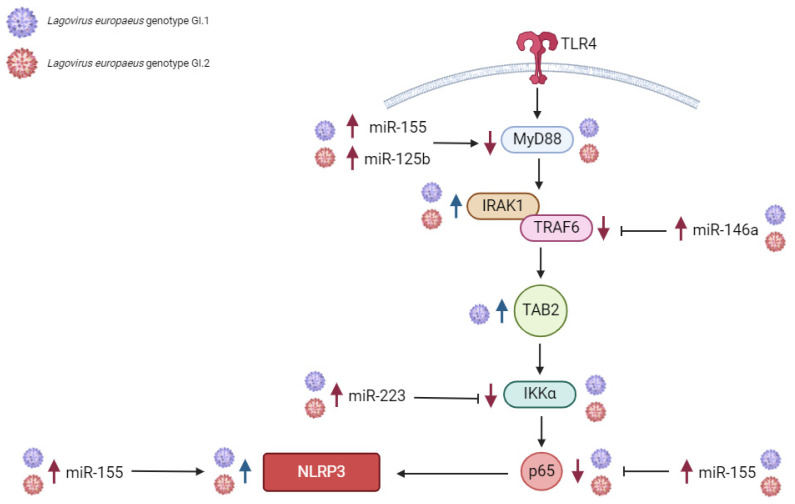
Contribution of miRs/target genes involved in the innate immune and inflammatory response pathway in the liver in *Lagovirus europaeus* infection. In the liver, increased miR-155 expression during *L. europaeus*/GI.1 and GI.2 genotypes inhibit *MyD88* and *p65* (*MyD88*—Spearman’s rank correlation Rho: −0.75, *p* = 0.013, *p65*—Spearman’s rank correlation Rho: −0.93, *p* < 0.001 for GI.1 and *MyD88*—Spearman’s rank correlation Rho: −0.93, *p* < 0.001, *p65*—Spearman’s rank correlation Rho: −0.7, *p* = 0.02 for GI.2). miR-155 induces *NLRP3* (Spearman’s rank correlation Rho: 0.98, *p* < 0.001 for GI.1 and Spearman’s rank correlation Rho: 0.8, *p* < 0.01 for GI.2). Increased expression of miR-146a inhibits *TRAF6* (Spearman’s rank correlation Rho: −0.91, *p* < 0.001 for GI.1 and Spearman’s rank correlation Rho: −0.81, *p* < 0.01 for GI.2). Enhanced expression of miR-223 correlates with reduced expression levels of *IKKα* (Spearman’s rank correlation Rho: −0.87, *p* = 0.001 for GI.1 and Spearman’s rank correlation Rho: −0.83, *p* = 0.003 for GI.2) and with *TLR4* despite a statistically significant increase in this mRNA (Spearman’s rank correlation Rho: −0.9, *p* < 0.001 for GI.1 and Spearman’s rank correlation Rho: −0.93, *p* < 0.001 for GI.2). Additionally, an increase miR-125b correlates with a decrease in *MyD88* (Spearman’s rank correlation Rho: −0.63, *p* = 0.048 for GI.1 and Spearman’s rank correlation Rho: −0.8, *p* = 0.004 for GI.2).

**Figure 8:**
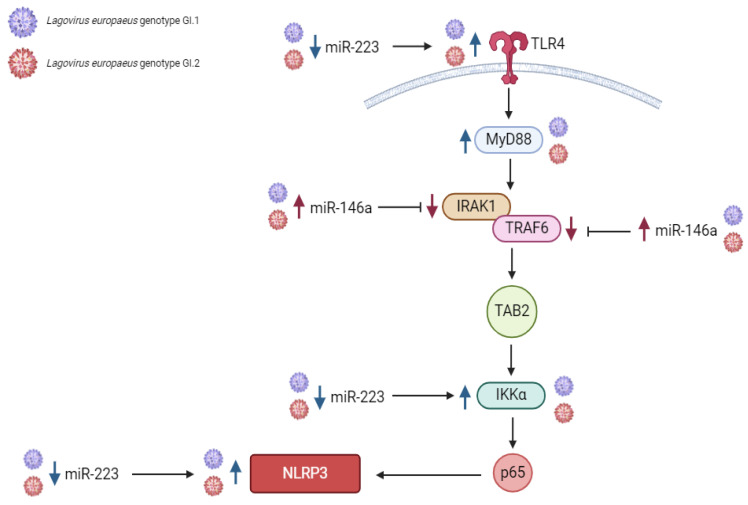
Contribution of miRs/target genes involved in the innate immune and inflammatory response pathway in the lungs in *Lagovirus europaeus* infection. In the lungs, reduced miR-223 expression levels results in increased expression of *TLR4* (Spearman’s rank correlation Rho: −0.7, *p* = 0.025 for GI.1 and Spearman’s rank correlation Rho: −0.95, *p* < 0.001 for GI.2), *IKKα* (Spearman’s rank correlation Rho: −0.87, *p* < 0.001 for GI.1 and Spearman’s rank correlation Rho: −0.92, *p* < 0.001 for GI.2) and *NLRP3* (Spearman’s rank correlation Rho: −0.69, *p* = 0.025 for GI.1 and Spearman’s rank correlation Rho: −0.92, *p* < 0.001 for GI.2). Increased miR-146a expression inhibits the *IRAK1* and *TRAF6* target genes (*IRAK1*—Spearman’s rank correlation Rho: −0.86, *p* = 0.001 for GI.1 and Spearman’s rank correlation Rho: −0.7, *p* = 0.02 for GI.2, *TRAF6*—Spearman’s rank correlation Rho: −0.63, *p* 0.048 for GI.1 and Spearman’s rank correlation Rho: −0.96, *p* < 0.001 for GI.2). In the case of the liver and kidney, we did not observe an inhibitory effect of miR-155 on the *TAB2* target gene during *L. europaeus*/GI.1 and GI.2 infection.

**Figure 9:**
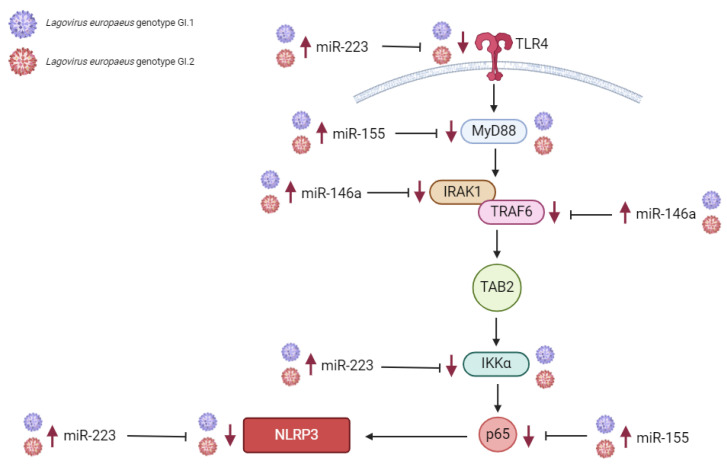
Contribution of miRs/target genes involved in the innate immune and inflammatory response pathway in the kidney in *Lagovirus europaeus* infection. In the kidney, an increase in miR-223 during infection of both virus genotypes inhibits *TLR4* (Spearman’s rank correlation Rho: −0.84, *p* = 0.002 for GI.1 and Spearman’s rank correlation Rho: −0.7, *p* = 0.02 for GI.2), *IKKα* (Spearman’s rank correlation Rho: −0.85, *p* = 0.0016 for GI.1 and Spearman’s rank correlation Rho: −0.92, *p* < 0.001 for GI.2) and *NLRP3* (Spearman’s rank correlation Rho: −0.91, *p* < 0.001 for GI.1 and Spearman’s rank correlation Rho: −0.95, *p* < 0.001 for GI.2). Upregulation of miR-155 correlates negatively with *MyD88* (Spearman’s rank correlation Rho: −0.75, *p* = 0.01 for GI.1 and Spearman’s rank correlation Rho: −0.92, *p* < 0.001 for GI.2) and *p65* (Spearman’s rank correlation Rho: −0.8, *p* = 0.005 for GI.1 and Spearman’s rank correlation Rho: −0.98, *p* < 0.001 for GI.2), resulting in inhibition of expression. MiR-146a inhibits the *IRAK1* (Spearman’s rank correlation Rho: −0.95, *p* < 0.001 for GI.1 and Spearman’s rank correlation Rho: −0.92, *p* < 0.001 for GI.2) and *TRAF6* (Spearman’s rank correlation Rho: −0.92, *p* < 0.001 for GI.1 and Spearman’s rank correlation Rho: −0.92, *p* < 0.001 for GI.2) target genes.

**Figure 10:**
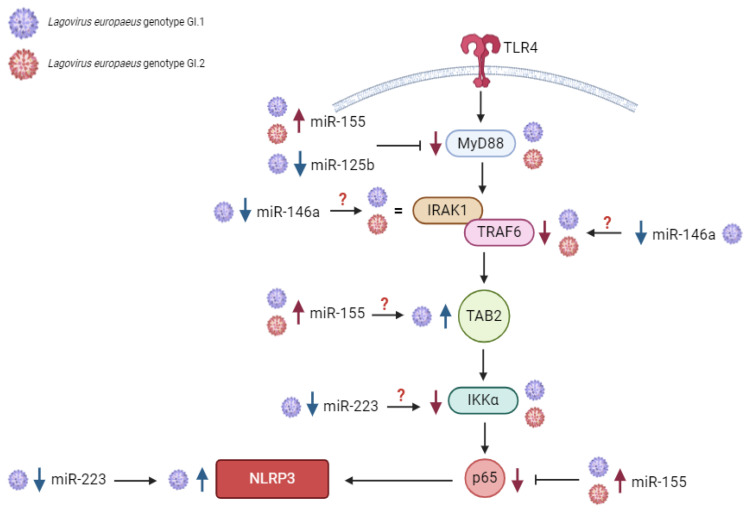
Contribution of miRs/target genes involved in the innate immune and inflammatory response pathway in the spleen in *Lagovirus europaeus* infection. In the spleen, the miR-155 expression increases during *L. europaeus*/GI.1 and GI.2 genotypes’ infections inhibit *MyD88* and *p65* (*MyD88*—Spearman’s rank correlation Rho: −0.81, *p* = 0.004, *p65*—Spearman’s rank correlation Rho: −0.98, *p* < 0.001 for GI.1 and *MyD88*—Spearman’s rank correlation Rho: −0.81, *p* = 0.004, *p65*—Spearman’s rank correlation Rho: −0.91, *p* < 0.001 for GI.2). Decreased expression of miR-223 correlates with upregulated expression level of *NLRP3* (Spearman’s rank correlation Rho: −0.95, *p* < 0.001 for GI.1). ?—Requires further research.

The contribution of miRs/target genes involved in the innate immune and inflammatory response pathway in *L. europaeus* infection is presented separately for each tissue (Figure 7, Figure 8, Figure 9 and Figure 10). Additionally, Spearman’s rank correlations for examined miRs and the mRNA in four tissues of rabbits during *L. europaeus*/GI.1 and GI.2 infection were described. Correlations are provided for statistically significant results (Figure 7, Figure 8, Figure 9 and Figure 10).

### 3.2. Proposed Inflammation Profiles in Response to L. europaeus Infection of GI.1 and GI.2 Genotypes

**Figure 11:**
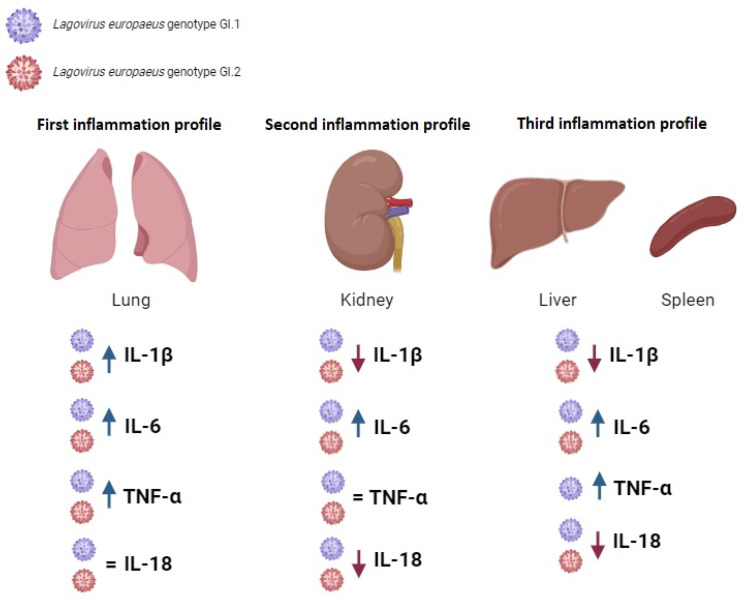
Inflammation profiles in response to *L. europaeus* infection of both the GI.1 and GI.2 genotypes.

Based on the inflammatory biomarkers, we distinguished three inflammatory profiles in the examined tissues (Figure 11):

(1) Pulmonary profile (increase three key acute phase cytokines- IL-1β, IL-6, TNF-α; IL-18-no change);
(2) Renal profile (increase: IL-6, decrease: IL-1β, IL-18; TNF-α -no changes);
(3) Liver and spleen profile (increase: IL-6, TNF-α; decrease: IL-1β, IL-18).

Distinct patterns of tissue cytokines may prove essential to guide the diagnosis and treatment of viral diseases, acute liver failure (ALF), and multi-organ failure (MOF) of viral etiology. In practice, plasma cytokine profiling is routinely used in patients with inflammation to define the pathophysiological phenotype, thereby playing a pivotal role in diagnosing and therapeutic decision making [58].

## 4. Materials and Methods

### 4.1. Ethical Statements

The experiment was carried out in the experimental animal facility of the Pomeranian Medical University (PUM) in Szczecin based on the consent obtained by the Local Ethical Committee for Animal Experiments in Poznań, Poland (no. 51/2022). Rabbits were maintained according to the European Union and national guidelines for animal experimentation. Additionally, during the experiment, the clinical conditions of the infected and healthy control rabbits were assessed, and clinical signs and mortality were recorded for the infected animals.

### 4.2. Viruses

The viruses used in the experiment have been prepared at the National Reference Laboratory for Rabbit Hemorrhagic Disease (RHD) and the Department of Foot and Mouth Disease, the National Veterinary Research Institute—State Research Institute in Zduńska Wola, Poland. To provoke infection in rabbits, two viruses were utilized: the *Lagovirus europaeus* genotype GI.1, variant GI.1a, which was designated as BBI (Poland, 2017; GenBank accession no. MG602005), and *Lagovirus europaeus* genotype GI.2 which was labeled as PIN (Poland, 2018; GenBank accession no. MN853660) [80]. Both viruses were titer-determined by the hemagglutination (HA) assay. The infectious titer of the *L. europaeus* GI.1 genotype inoculum (1 mL) was determined to be 0.5 u/mL (1 HA unit corresponds to 10^4^ particles per mL), and for *L. europaeus* GI.2 genotype to be 2.048 u/mL (1 HA unit corresponds to 10^4^ particles per mL).

### 4.3. Experimental Models

The study was performed on 30 European rabbits. *Oryctolagus cuniculus*—Crl:KBL (NZW)/052 purchased from AnimaLab Limited Liability Company (branch in Poland, Poznań). The rabbits were 6 months old, with body weights of 4.0–4.5 kg, and of both sexes (50:50 ratio). After the animals were delivered to the university’s experimental facility, there was a 3-week adaptation period. The experiment was conducted in accordance with the European Union Directive with regard to temperature and humidity, as well as the lighting and size of cages for animals [81], and ARRIVE guidelines [82,83]. The autonomous air conditioning system maintained a temperature of 22 °C (±1 °C) with humidity levels between 50% and 60%. The system facilitated 15–20 air changes per hour, ensuring a controlled environment. The rooms were equipped with artificial lighting, automatically controlled to provide 12 h of light followed by 12 h of darkness, supplemented with red night lighting. Food and water were available to the animals *ad libitum*. The animals were randomly divided into three groups for the study. The experimental animals were infected by intramuscular injection of the 1 mL selected virus—the *L. europaeus*/GI.1 genotype (named BBI strain, Poland 2017) for group 1, and the *L. europaeus*/GI.2 genotype (PIN strain, Poland 2018) for group 2, while the control group was injected with a form of PBS (phosphate-buffered saline) as a placebo. The onset of severe symptoms of rabbit hemorrhagic disease was considered the terminal moment of the experiment. Animals qualified for euthanasia were anesthetized by intravenous administration of the preparation ketamine 35–50 mg/kg, xylazine 5–10 mg/kg, followed by administration of the cardiac arrest-inducing preparation sodium pentobarbital (at 240 mg/kg).

### 4.4. Tissue Sample Collection

Tissue samples for the study (liver, lung, spleen, and kidney) were obtained from infected rabbits (*n* = 20) immediately post-mortem and from healthy rabbits (*n* = 10) after euthanasia. All tissues were washed in cold PBS, placed in liquid nitrogen, and stored at −80 °C until total RNA extraction.

### 4.5. Selection and In-Silico Prediction of miRs Target Genes Involved in the Innate Immune and Inflammatory Response in Oryctolagus cuniculus

In the first stage, miRs involved in innate immune and inflammatory responses were selected [24,25]. At this stage, the miRTarBase database [84] was used to select miRs using various search strategies (by miRs, target gene, pathway/process, validated methods, and disease). The criterion for selecting miRs was the validation method: strong evidence and previous literature data [24,25,46]. *Homo sapiens*’ miRs with a described role in the innate immune and inflammatory response (miR-155, miR-146a, miR-223, miR-125b) were selected [24,25,46,84]. In the next stage, the miRTarBase database [84] and miRDB database [85] were used to select target genes (for chosen miRs) previously validated by RT-qPCR, Western blot, or a reporter assay in other species (validation methods: strong evidence). Next, the set of genes was used to conduct a gene ontology (GO) analysis via a GO enrichment analysis powered by protein annotation through evolutionary relationships [86]. The analysis included analysis type: PANTHER overrepresentation test; reference list: all *Homo sapiens’* genes in the database; annotation data set: GO biological process complete; test type: Fisher’s exact; and correction: calculate false discovery rate (FDR). From all the processes with FDR *p* < 0.05, those correlated with immune response, innate immune response, inflammatory response, liver diseases, and multi-organ dysfunction in humans and animals were used for further steps. The 3′-UTR sequences of the *Oryctolagus cuniculus* genes involved in the selected processes were assessed to determine if they featured binding sites for miR-155, miR-146a, miR-223, and miR-125b using the TargetScan database [87]. In the next stage, to verify the importance of miR-155, miR-146a, miR-223, and miR-125b in *L. europaeus* infection, an in-silico analysis of putative target genes was conducted. Due to the inability to use one database to demonstrate the miR–mRNA interactions in *Oryctolagus cuniculus*, the following approach was selected: (i) mature sequences of these miRs in *Oryctolagus cuniculus* and *Homo sapiens* were compared, and no differences were found. We decided to use the miRTarBase [84], which lists genes with validated miR–mRNA interactions by RT-qPCR or luciferase assays in *Homo sapiens*. Finally, four groups were created containing 110 for miR-155, 90 for miR-146a, 100 for miR-223, and 50 for miR-125b; (ii) there was an attempt to determine the processes related to RHD that miR-155, miR-146a, miR-223, and miR-125b might regulate. For this purpose, a GO analysis was conducted on the putative target genes for every miR separately. Thus, 772 processes for miR-155, 250 processes for miR-146a, 300 processes for miR-223, and 50 processes for miR-125b were identified. These groups’ processes correlated with RHD pathogenesis, liver diseases, ALF, and MOF and were subsequently chosen for further analysis: 22 for miR-155, 20 for miR-146a, 20 for miR-223, and 18 for miR-125b. At this step, all analyses were performed based on miR–mRNA interactions in *Homo sapiens*. The TargetScan database confirmed whether these regulations might also occur in *Oryctolagus cuniculus*. This tool enabled us to verify if the predicted binding sites were conserved in *Oryctolagus cuniculus*. Genes engaged in RHD, ALF, and MOF processes were selected. Each miR–3′-UTR interaction was checked independently. The TargetScan analysis revealed that 63 out of 159 genes for ocu-miR-155, 25 out of 50 genes for ocu-miR-146a, 20 out of 80 genes for ocu-miR-223, and 11 out of 40 genes for ocu-miR-125b, have binding sites in 3′-UTR in *Oryctolagus cuniculus* genes. Selected miRs/target genes are presented in Table 2.

### 4.6. miRs and mRNA Isolation from Tissues

The total RNA, encompassing miRs, was extracted from 50 mg of each tissue sample of the infected and healthy rabbits using the miRNeasy Mini Kit (Qiagen, Hilden, Germany). Isolation of total RNA was performed following the manufacturer’s protocol. Samples were mechanically homogenized in 700 µL of QIAzol Lysis Reagent, followed by a 5 min incubation at room temperature. Chloroform (140 µL) was added, and the mixture was shaken. After a 3 min incubation, the samples were centrifuged at 12,000× *g* for 15 min at 4 °C. The upper aqueous phase was carefully transferred to a new tube, and RNA was precipitated by adding 1.5 volumes of 100% ethanol. The mixture was then applied to an RNeasy Mini column, and centrifugation was performed at 8000× *g* for 15 s. This step was repeated for the entire sample. Subsequently, the column was washed with 700 µL of Buffer RWT, followed by two washes with 500 µL of Buffer RPE; the second included a 2 min centrifugation to ensure membrane dryness. Finally, RNA was eluted in 50 µL of RNase-free water after centrifugation at 9000× *g* for 1 min. RNA concentration and quality were assessed using a NanoDrop 2000 spectrophotometer (Thermo Fisher Scientific, Waltham, MA, USA).

### 4.7. miRs Polyadenylation and Reverse Transcription Reaction

cDNA synthesis was performed by the reverse transcription (RT) reaction using a miRCURY LNA RT Kit (Qiagen, Hilden, Germany). The total volume of 10 µL of the reaction mixture contained 2 µL of 5× miRCURY RT Reaction Buffer, 1 µL of miRCURY RT Enzyme Mix, 5 µL of RNase-free water and 2 µL of template RNA. Total RNA at a concentration of 5 ng/µL was used for the RT reaction. The reaction and temperature profile were according to the manufacturer’s recommendation with the following steps: reverse transcription step by incubation at 42 °C for 60 min, heat inactivation of the reverse transcriptase for 5 min at 95 °C, and immediate cooling to 4 °C. The obtained cDNA samples were stored at −20 °C.

### 4.8. mRNAs Polyadenylation and Reverse Transcription Reaction

The RT reaction to synthesize the cDNA from mRNA was carried out using the RevertAid First Strand cDNA Synthesis Kit (Thermo Fisher Scientific, USA). To increase the efficiency of the reaction oligo(dT), random hexamers were used. RT reaction was performed in the mixture with a final volume of 20 µL containing 1 µL of Oligo(dT) Primer and Random Hexamer primer, 4 µL of 5× Reaction Buffer, 1 µL of RiboLock RNase Inhibitor, 1 µL of RevertAid M-MuL V RT (200 U/µL), 4 µL of total RNA template of concentration 200 ng/µL and 8 µL of RNase-free water to replenish the mixture volume. The reaction conditions and temperature profile were determined based on the manufacturer’s instructions and were as follows: incubation for 5 min at 25 °C followed by 60 min at 42 °C, then heating at 70 °C for 5 min in order to termination of the reaction, and immediate cooling to 4 °C. The obtained cDNA samples were stored at −20 °C.

### 4.9. Quantification of miRs in Tissue Samples Using Quantitative Real-Time PCR and Data Analysis

The levels of miRs (Table 3) were measured using the quantitative real-time PCR (qPCR) reaction. The expression of miRs (along with miR-103a-3p, which was used as an endogenous control) in tissues samples were measured using the miRCURY LNA miRNA PCR Assay (Qiagen, Hilden, Germany) and the miRCURY LNA SYBR Green PCR Kit, according to the manufacturer’s instructions. The reaction mixture consisted of 5 µL of 2× miRCURY SYBR Green Master Mix, 0.05 µL of ROX Reference Dye, 1 µL of specific PCR primer mix, and 1 µL of RNase-free water. Three µL of 60× diluted template cDNA was added to the reaction mixture. The final reaction volume was 10 µL. The thermal cycle conditions were as follows: 95 °C for 2 min for the PCR initial heat activation, 40 cycles of denaturation 95 °C for 10 s and annealing 56 °C for 1 min, and at the end of the reaction, a melting curve analysis was performed in the temperature range of 60–95 °C. Fluorescence data were analyzed using Quant Studio 5 Real-Time PCR System (Applied Biosystems, Waltham, MA, USA) and the expression of miRs were calculated using the 2^−ΔΔCt^ method of relative quantification.

### 4.10. Quantification of mRNAs in Tissue Samples Using Quantitative Real-Time PCR and Data Analysis

Relative expression mRNA of selected genes (Table 4) was assessed by qPCR using the Quant Studio 5 Real-Time PCR System (Applied Biosystems, United States) and the HOT FIREPol^®^ EvaGreen^®^ qPCR Supermix, 5× (Solis BioDyne, Tartu, Estonia) according to manufacturer’s recommendations. The reaction mixture was 20 µL. It consisted of 4 µL of HOT FIREPol EvaGreen qPCR Supermix (5×), 0.4 µL of forward and reverse primers of concentration 10 µM and a variable volume of template cDNA of 10 ng/µL concentration and RNase-free water depending on the primer. The reaction was carried out according to the following temperature–time profile: initial activation by incubation at 95 °C for 12 min, 40 cycles of denaturation at 95 °C for 15 s, annealing at a temperature adapted to the primers (Table 4) for 20–30 s (depending on length product) and extension at 72 °C for 20–30 s. Specific primers for selected target genes for qPCR reaction were designed using the computational tools online Primer-BLAST (2024) [99] and Beacon Designer (2024) [100]. To validate the designed primers, a temperature gradient PCR was performed using the Color OptiTaq PCR Master Mix (2×) kit (Euryx, Gdańsk, Poland), followed by agarose gel electrophoresis. The PCR reaction mixture was 50 µL and consisted of 25 µL Color OptiTaq PCR Master Mix (2×), 0.25 µL of forward and reverse primers of 10 mM concentration, 23.5 µL RNase-free water, and 1 µL of CDNA template of 0.2 µg/µL concentration. The reaction was carried out according to the following temperature–time profile: initial denaturation at 94 °C for 2 min, 30 cycles of denaturation at 94 °C for 15 s, the annealing temperature was carried out in a temperature gradient (temperature range of 52–62 °C) for 30 s, and extension at 72 °C 1 min. Final extension was at 72 °C for 7 min. After the reaction was completed, the samples were cooled to 4 °C. This process enabled determination of the optimal annealing temperature for the primers and to check for the formation of non-specific products. After primer validation, optimization of the real-time PCR reaction was performed. Optimization approach involved sequentially adjusting primer sequences, annealing temperatures, primer concentrations, and a range of cDNA concentrations for each gene tested. The calibration method was used to obtain a standard curve. Using the optimal annealing temperature and primer concentration for each primer pair, serial dilutions of the same cDNA were performed (1:2, 1:4, 1:8, 1:16, and 1:32) from an initial concentration of 10 ng/μL, as recommended by the manufacturer (HOT FIREPol^®^ EvaGreen^®^ qPCR Supermix, 5×; Solis BioDyne, Estonia). It was observed that different primer pairs required different optimal cDNA concentration ranges for each gene to achieve the highest coefficient of determination (R^2^) and optimal efficiencies (100% ± 5%). Additionally, the specificity of the primers was confirmed by melting curve analysis at the end of the reaction real-time PCR. Fluorescence data were analyzed using a real-time PCR system. The amount of target, normalized to an endogenous reference gene 18S and relative to the expression levels in healthy controls, was determined using the 2^−ΔΔCt^ formula. A melting curve analysis was performed each time.

### 4.11. Statistical Analysis

Statistical analysis was performed using STATISTICA PL Version 13 (StatSoft, Palo Alto, CA, USA). The data were evaluated as a mean ± standard error of the mean (SEM) for continuous variables. The normal distribution of the analyzed variables was tested using the Shapiro–Wilk test. For data analysis the Student’s *t*-test was performed for data with a normal distribution, and for data with a non-parametric distribution, the Mann–Whitney U test was performed. To determine possible changes in all miRs or mRNAs, the one-way ANOVA was performed or Kruskal–Wallis test depending on the obtained distribution. Additionally, correlation analysis was performed using the test non-parametric Spearman’s rank method. Spearman’s rank correlation coefficient (R) was also calculated to measure the statistical dependence between expression of miRs and the expression of their target genes. In all statistical tests, the results were considered statistically significant if the *p*-value did not exceed 5% (*p* ≤ 0.05). 

## 5. Conclusions

Our report is the first to present the regulatory effect of miRs on innate immune and inflammatory response genes in rabbits infected with *L. europaeus*/GI.1 and GI.2 genotypes in four tissues (liver, lung, kidney, and spleen).

Our research provides new data for understanding the pathogenesis of rabbit hemorrhagic disease caused by *L. europaeus* and understanding the molecular regulation of the innate immune and inflammatory response.

The main regulators of the innate immune and inflammatory response in *L. europaeus*/GI.1 and GI.2 infection, as well as RHD, are miR-155, miR-223, and miR-146a. miR-125 expression was highly limited and affected only the liver and spleen. We have shown that during infection with *L. europaeus*/GI.1 and GI.2/RHD, (1) miR-155—has both pro- and anti-inflammatory effects in the liver and anti-inflammatory effects in the kidneys and spleen; (2) miR-146a has anti-inflammatory effects in the liver, lungs and kidneys; (3) miR-223 has anti-inflammatory effects in all tissues; (4) however, miR-125b has anti-inflammatory effects only in the liver. In each case, such an effect may be a determinant of the pathogenesis of RHD. Our research shows that miRs may regulate three innate immune and inflammatory response pathways in *L. europaeus* infection: (1) TLR4-MyD88 signaling pathway; (2) NF-ĸB signaling pathway (p65); and (3) NRLP3 inflammasome pathway. However, the result of this regulation may be influenced by the tissue microenvironment, as observed in studies.

Our report is the first to show the expression profile of inflammatory biomarkers (IL-1β, IL-6, TNF-α, IL-18) in four tissues after infection with *L. europaeus*—with two genotypes simultaneously. Our research shows that infection of rabbits with *L. europaeus*/GI.1, and GI.2 genotypes causes an overexpression of two critical acute phase cytokines—*IL-6* in all examined tissues and *TNF-α* (in the liver, lungs, and spleen). Cytokine *IL-1β* was highly expressed only in the lungs after *L. europaeus* infection. These facts indicate a strong and rapid involvement of the local innate immune and inflammatory response in *L. europaeus* infection for both genotypes (GI.1 and GI.2) and in the pathogenesis of RHD. Profile biomarkers of inflammation in rabbits infected with *L. europaeus* by both genotypes are similar regarding the nature of changes but are different for individual tissues. Therefore, we propose three inflammation profiles for *L. europaeus* infection (pulmonary, renal, and liver and spleen).

The results of our research also have diagnostic (search for potential molecular biomarkers of inflammation/disease) and therapeutic potential (modulation of miR-dependent pathways, e.g., NF-ĸB and NLRP3 inflammasome) in the course of acute liver failure (ALF) and organ dysfunction in multi-organ failure (MOF) of a viral etiology that we encounter during *Lagovirus europaeus* infection.

## Figures and Tables

**Table 1:** Comparison of expression biomarkers of inflammation in four tissues of rabbits infected with *Lagovirus europaeus*/GI.1 and GI.2 genotypes.

Tissues	Proinflammatory Cytokines Determined at the mRNA Level			
Gene	*IL-1β*	*IL-6*	*TNF-α*	*IL-18*
**Liver**				
Control vs. GI.1	↓ ×3.6	↑ ×8.0	↑ ×5.7	↓ ×46.8
Control vs. GI.2	↓ ×3.1	↑ ×6.8	=	↓ ×31.0
GI.1 vs. GI.2	=	=	↑ ×6.4	=
**Lung**				
Control vs. GI.1	↑ ×3.1	↑ ×7.7	↑ ×3.1	=
Control vs. GI.2	↑ ×6.7	↑ ×12.3	↑ ×2.4	=
GI.1 vs. GI.2	=	=	=	=
**Kidney**				
Control vs. GI.1	↓ ×3.6	↑ ×8.5	=	↓ ×27.5
Control vs. GI.2	↓ ×2.0	↑ ×261.0	=	↓ ×3.3
GI.1 vs. GI.2	=	↓ ×30.0	↓ ×2.1	↓ ×8.3
**Spleen**				
Control vs. GI.1	↓ ×5.5	↑ ×997.0	↑ ×1.9	↓ ×3.0
Control vs. GI.2	↓ ×1.9	↑ ×282.0	=	↓ ×5.1
GI.1 vs. GI.2	↑ ×5.2	=	=	↑ ×1.7

Explanations: ↑—increase in expression; ↓—decrease in expression; and = no statistical significance.

**Table 2:** Selected studied miRs/target genes involved in innate immune and inflammatory responses.

miRs	Target Genes	Gene Product	Reference Used to Select Target Gene
miR-155	*MyD88*	Myeloid differentiation primary response protein MyD88	[46,88,89]
	*TAB2*	TGF-beta-activated kinase 1 and MAP3K7-binding protein 2	[46,90,91]
	*p65* (subunit of NF-κβ)	Transcription factor p65	[46,92,93]
	*NLRP3 inflammasome*	NACHT, LRR, and PYD domains containing protein 3	[46]
miR-146a	*IRAK1*	Interleukin-1 receptor-associated kinase 1	[25,46,94]
	*TRAF6*	TNF receptor-associated factor 6	[25,46,94]
miR-223	*TLR4*	Toll-like receptor 4	[46,68,95]
	*IKKα*	Inhibitor of nuclear factor kappa-B kinase subunit alpha	[46,67,96]
	*NLRP3*	NACHT, LRR, and PYD domains-containing protein 3	[46,97]
miR-125b	*MyD88*	Myeloid differentiation primary response protein MyD88	[50,98]

**Table 3:** Sequences of the tested microRNAs (miR) of *Oryctolagus cuniculus* (ocu).

miRs	Sequences
**miRs tested**	
ocu-miR-155-5p	5′UUAAUGCUAAUCGUGAUAGGGGUU3′
ocu-miR-146a-5p	5′UGAGAACUGAAUUCCAUGGGUUG3′
ocu-miR-223-5p	5′CGUGUAUUUGACAAGCUGAGUUG3′
ocu-miR-125b-5p	5′UCCCUGAGACCCUAACUUGUGA3′
**reference miRs**	
ocu-miR-103a-3p	5′AGCAGCAUUGUACAGGGCUAUGA3′

**Table 4:** qPCR primers used in the study to target genes expression.

Gene	GenBank Accession No.	Primers		Ta (°C)	Amplicon Length (bp)	Tm of the Amplification Products (°C)
*MyD88*	XM_002723869.4	Forward Revers	5′-CCCCAGCGACATGCAGTTTG-3′ 5′-TTCTGATGGGCACCTGGAGAG-3′	61	227	90.8
*TAB2*	XM_051836813.1	Forward Revers	5′-ACCTCCAGCAGTTCCTCTTC-3′ 5′-TCATCTCCTGTGGTGGCATT-3′	60	152	83.5
*p65*	XM_051827970.1	Forward Revers	5′-CCCTTCCAAGTGCCCATAGA-3′ 5′-CCTCTTTCTGCACCTTGTCG-3′	60	250	91.5
*NLRP3*	MK829787.1	Forward Revers	5′-TGTCTCACGTCCAGCTTTTG-3′ 5′-AGCCAGAGTCTGCGAATGTT-3′	60	161	87.5
*IRAK1*	XM_051837494.1	Forward Revers	5′-GGACTTTGCTGGCTACTGTG-3′ 5′-CAGGAGGACGTTGGAACTCT-3′	60	229	89.9
*TRAF6*	XM_002709054.4	Forward Revers	5′-ACGGGGAACCTTTCTGGCTC-3′ 5′-TGTGGCCTGCATCCCTTATTG-3′	61	187	86.4
*TLR4*	NM_001082732.2	Forward Revers	5′-TTTCACACGGCCACTGCTG-3′ 5′-ATTGGGAACGACCTCCACAC-3′	61	142	81.4
*IKKα*	XM_002718612.4	Forward Revers	5′-GGTAACTCCTCAAGATGGGGAC-3′ 5′-TGCCCTGTTCCTCATTTGCT-3′	60	107	78.7
*IL-1β*	NM_001082201.1	Forward Revers	5′-GGTGTTGTCTGGCACGTATG-3′ 5′-TTGGGGTCTACACTCTCCAG-3′	60	210	84.0
*IL-6*	NM_001082064.2	Forward Revers	5′-GGCGGTGAATAATGAGACCTG-3′ 5′-ATGAAGTGGATCGTGGTCGT-3′	60	276	87.3
*TNF-α*	NM_001082263.1	Forward Revers	5′-CGTAGTAGCAAACCCGCAAG-3′ 5′-TGATGGCAGAGAGGAGGTTG-3′	60	245	91.3
*IL-18*	NM_001122940.1	Forward Revers	5′-TGTATAGAAAATGCACCCCAGAC-3′ 5′-TCTTTCTGTCCTGCGAGATGT-3′	60	221	80.0
*18S*	NR_033238.1	Forward Revers	5′-ATCAGATACCGTCGTAGTTC-3′ 5′-TTCCGTCAATTCCTTTAAG-3′	60	167	88.0

## Data Availability

The original contributions presented in the study are included in the article; further inquiries can be directed to the corresponding author.
